# The cryptic lncRNA-encoded microprotein TPM3P9 drives oncogenic RNA splicing and tumorigenesis

**DOI:** 10.1038/s41392-025-02128-8

**Published:** 2025-01-27

**Authors:** Kun Meng, Yuying Li, Xiaoyi Yuan, Hui-Min Shen, Li-Ling Hu, Danya Liu, Fujin Shi, Dandan Zheng, Xinyu Shi, Nengqiao Wen, Yun Cao, Yun-Long Pan, Qing-Yu He, Chris Zhiyi Zhang

**Affiliations:** 1 MOE Key Laboratory of Tumor Molecular Biology and State Key Laboratory of Bioactive Molecules and Druggability Assessment, Institute of Life and Health Engineering, College of Life Science and Technology, Jinan University, Guangzhou, 510632 China; 2 Xiangyang Central Hospital, Affiliated Hospital of Hubei University of Arts and Science, Hubei Province 441100 Xiangyang, China; 3 Department of Obstetrics and Gynecology, The First Affiliated Hospital, Sun Yat-sen University, Guangzhou, 510080 China; 4 Department of Pathology, State Key Laboratory of Oncology in South China, Sun Yat-sen University Cancer Center, 510060 Guangzhou, China; 5 The First Affiliated Hospital of Jinan University, Jinan University, Guangzhou, 510632 China

**Keywords:** Urological cancer, Kidney diseases

## Abstract

Emerging evidence demonstrates that cryptic translation from RNAs previously annotated as noncoding might generate microproteins with oncogenic functions. However, the importance and underlying mechanisms of these microproteins in alternative splicing-driven tumor progression have rarely been studied. Here, we show that the novel protein TPM3P9, encoded by the lncRNA tropomyosin 3 pseudogene 9, exhibits oncogenic activity in clear cell renal cell carcinoma (ccRCC) by enhancing oncogenic RNA splicing. Overexpression of TPM3P9 promotes cell proliferation and tumor growth. Mechanistically, TPM3P9 binds to the RRM1 domain of the splicing factor RBM4 to inhibit RBM4-mediated exon skipping in the transcription factor TCF7L2. This results in increased expression of the oncogenic splice variant TCF7L2-L, which activates NF-κB signaling *via* its interaction with SAM68 to transcriptionally induce RELB expression. From a clinical perspective, TPM3P9 expression is upregulated in cancer tissues and is significantly correlated with the expression of TCF7L2-L and RELB. High TPM3P9 expression or low RBM4 expression is associated with poor survival in patients with ccRCC. Collectively, our findings functionally and clinically characterize the “noncoding RNA”-derived microprotein TPM3P9 and thus identify potential prognostic and therapeutic factors in renal cancer.

## Introduction

Approximately 2% of the human genome can potentially encode proteins.^1^ The remaining transcripts were previously considered noncoding RNAs, such as lncRNAs, pri-miRNAs and circRNAs.^2^ Currently, due to the advances of multiomics profiling *via* deep sequencing techniques, including ribosomal mapping and computational biology approaches, small open reading frames (sORFs) in noncoding RNAs have been found to be translated into microproteins or small peptides that perform crucial functions in human cancers.^3–5^ According to the ENCODE database, approximately 17,000 lncRNAs, defined as transcripts of >200 nucleotides, theoretically exist in the human genome.^6^ A hidden proteome encoded by 3,300 lncRNAs was discovered, and 308 lncRNA-encoded novel proteins were detected *via* shotgun proteomics.^7^ Ribosome footprint profiling combined with lncRNA-Seq identified 12 lncRNAs with coding potential in colorectal cancer.^8^ The Ribo-TIS Hunter (Ribo-TISH) revealed 758 cryptic lncRNA-encoded open reading frames (ORFs) in breast cancer.^9^

Increasing interest has been given to exploring the roles of lncRNA-encoded microproteins in tumorigenesis and their clinical implications.^10,11^ For instance, the LINC00673-encoded RASON binds to *KRAS*^*G12D/V*^ to inhibit GTP hydrolysis and antagonize the effects of EGFR inhibitors.^12^ The lncRNA-derived SMIM30 promotes hepatocellular carcinoma progression by facilitating SRC/YES1 membrane anchoring, thereby activates the MAPK pathway.^13^ In medulloblastoma, the microprotein ASDURF enhances cell survival through its interaction with the prefoldin-like chaperone complex.^14^ SMIMP binds to SMC1A to modulate the formation of the cohesin complex and suppress the expression of cell cycle genes in colorectal cancer.^15^ Additionally, GT3-INCP, encoded by LINC00992, interacts with GATA3 to promote breast cancer.^9^ While it is well-established that microproteins encoded by non-coding RNAs play critical roles in various tumor processes, only a limited number of lncRNA-encoded microproteins have been reported in the context of renal cell carcinoma (RCC) tumorigenesis and progression.^16^ Nonetheless, some lncRNA-derived microproteins have been implicated in RCC tumor progression. For example, MIAC cooperates with AQP2 to inhibit EGFR expression and exhibits anti-tumor activity.^17^ LINC00887-encoded ACLY-BP protects ACLY from ubiquitin-mediated degradation to promote tumor progression.^18^ Additionally, the LINC00493-encoded microprotein SMIM26 exhibits anti-metastatic activity *via* interaction with the AGK-SCL25A11 complex.^19^

Systematic functional characterization of these cryptic microproteins may uncover more important biomolecules embedded within the dark proteome, enhancing our understanding of the coding potential of RNAs in the human genome. Advances in techniques such as ribosome profiling, proteomics, computational algorithms, and high-resolution mass spectrometry have revealed the existence of these previously overlooked microproteins, which originate from translation events beyond canonical coding sequences.^3,20^ MS-based proteomics remains the gold standard for the identification and characterization of proteins.^3,21^ However, the cellular functions of many identified non-canonical CDSs and their peptide products remain largely unexplored.

In the present study, we conducted a large-scale screening of lncRNA-encoded proteins (LEPs) in pan-cancer through a combination of translatomics and proteomics analysis and identified a lncRNA-derived microprotein, TPM3P9, which exhibits widespread expression across human cancers. Further investigations demonstrated the clinical significance and biological function of TPM3P9 in clear cell renal cell carcinoma (ccRCC), revealing that TPM3P9 promotes tumor growth by enhancing the alternative splicing of oncogenic RNAs. These findings not only clinically and functionally characterize TPM3P9 but also suggest its potential as a therapeutic target in the management of ccRCC. Moreover, our study highlights the critical role of non-canonical ORF translation in RCC, providing a strong rationale for including these ORFs in future research aimed at discovering new cancer targets.^14^ The identification of novel lncRNA-encoded peptides that modulate critical signaling pathways in RCC highlights the need for further exploration of these non-canonical proteins to enhance our understanding and treatment of renal cell carcinoma.

## Results

### Cryptic translation of microproteins hidden in lncRNAs in human cancers

**Fig. 1:**
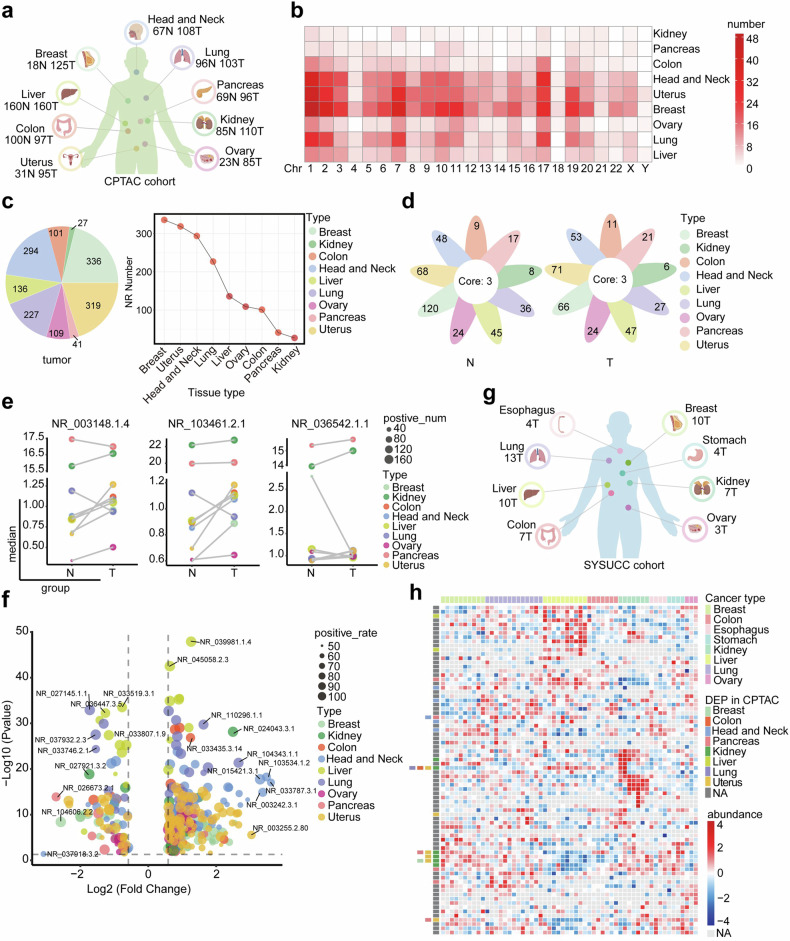
Cryptic translation of microproteins hidden in lncRNAs in human cancers. **a** Schematic representation showing an overview of pan-cancer types and their distributions in the CPTAC cohort (Created with BioRender.com). **b** The chromosomal distribution of LEPs identified in the CPTAC cohort. LEPs expressed in >30% of the tumor tissues or non-tumor tissues within a specific sample type were considered to be identified. **c** The number of LEPs identified in tumor tissues for each sample type. LEPs identified in 30% of the tumor samples were considered to be expressed in the tumor samples. **d** Flower plot. The petals represent the unique LEPs identified for each cancer type, and the core represents LEPs identified in all cancer types. **e** The expression levels of the three LEPs identified in all nine cancer types, with the size of the dot indicating positive rates and the color representing the cancer type. **f** Differentially expressed LEPs between tumor and non-tumor samples in the CPTAC cohort. Volcano plot showing the LEPs with |fold change | ≥ 1.5 and *p* < 0.05. **g** Schematic representation showing an overview of pan-cancer types and their distributions in the SYSUCC cohort (Created with BioRender.com). **h** Heatmap showing the LEPs identified in the SYSUCC cohort. LEPs expressed in >30% of the samples within a cancer type were considered to be identified

In our previous studies, we established the ribosome-nascent chain complex sequencing (RNC-seq) technique to determine that 3300 lncRNAs were bound to ribosomes with active translation elongation in nine cancer cell lines.^7,19,22^ In this study, proteomics data from the Clinical Proteomic Tumor Analysis Consortium (CPTAC), the Cancer Cell Line Encyclopedia (CCLE) and SYSUCC databases were used to screen candidate novel proteins of interest and conducted functional studies in renal cancer (Supplementary Fig. 1). Here, we acquired proteomic data from >1000 patients with nine types of malignant tumors in CPTAC (Fig. 1a). We then matched the 3300 translating lncRNAs to the CPTAC proteomic data, and identified 777 lncRNA-encoded proteins (LEPs) (Data S1), the lengths of LEPs are primarily distributed within the 50–200 amino acid (aa) range in tumor tissues (Supplementary Fig. 2a). These LEPs were distributed across various chromosomes (Fig. 1b and Data S2). Most LEPs were detected in over half of the kidney cancer samples but in fewer than 50% of lung cancer and head and neck cancer samples (Supplementary Fig. 2b and Data S3). The absolute numbers and the positive rates of LEPs identified in tumor tissues were noticeably different in human cancers (Fig. 1c, Supplementary Fig. 2c, d and Data S3). Proteins uniquely expressed in specific cancer types are listed in Fig. 1d and Data S4. Three proteins were found to be universally expressed in all tested cancer types and were differentially expressed between tumor and non-tumor tissues (Fig. 1e and Data S5). The expression of all the LEPs was further compared in nine types of cancer (Supplementary Fig. 2e and Data S6). LEPs expressed in >50% of the cases were subjected to differential expression analysis. The results indicated that LEPs were more frequently upregulated than downregulated in cancer tissues (Fig. 1f and Data S7). This may indicate that the expression of LEPs can be induced by oncogenic stress.

To validate the expression profile of LEPs in human cancers, we further analyzed LEP profiling in both CCLE (Supplementary Fig. 3a, Data S8) and cancer cell lines preserved in our laboratory (Supplementary Fig. 3b, Data S9). The results consistently showed that LEPs were widely present in cancer cell lines. Additionally, we collected postsurgical samples from 58 patients with eight types of solid cancers from SYSUCC (Fig. 1g). Using the same strategy employed in the CPTAC cohort, we identified 78 LEPs that were expressed in these 58 clinical cases (Fig. 1h, Supplementary Fig. 3c, Data S10 and S11). Among the 78 LEPs, several with differential expression between tumor and non-tumor tissues were also found in the CPTAC cohort. Specifically, kidney cancer showed a significant profile of LEPs expression, as compared with other cancers (Fig. 1h). This may suggest the deep involvement of LEPs in the progression of kidney cancer.

### The new protein TPM3P9 is upregulated in ccRCC, and its upregulation is correlated with poor prognosis

**Fig. 2:**
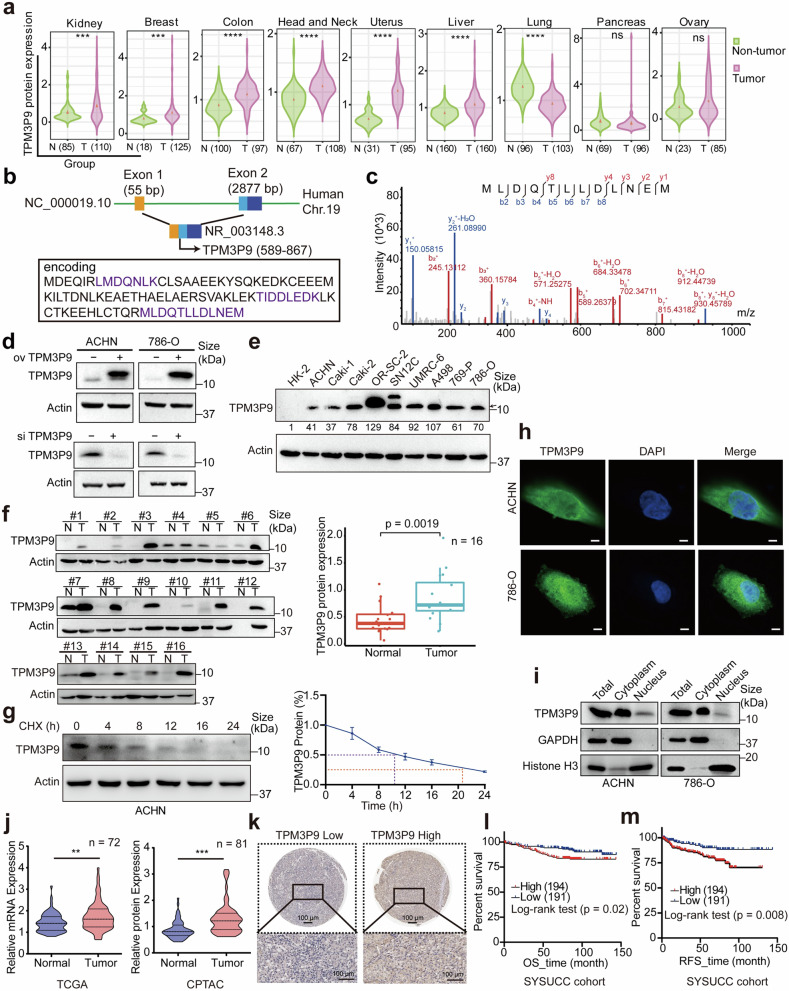
The novel microprotein TPM3P9 is upregulated in ccRCC, and its upregulation is correlated with poor prognosis. **a** Violin plot showing the expression levels of the lncRNA-TPM3P9 encoded microprotein TPM3P9 in pan-cancer and non-tumorous tissues. ***p < 0.001; ****p < 0.0001; ns, nonsignificant. **b** Schematic representation of the microprotein TPM3P9 translated from lncRNA-TPM3P9, with the transcript NR_003148.3. Unique peptides are visually represented in purple. **c** The representative unique peptide of TPM3P9 was identified by shotgun mass spectrometry of ccRCC cell lines. **d** Verification of endogenous and exogenous TPM3P9 expression in ccRCC cells with TPM3P9 overexpression or knockdown, using a TPM3P9-specific antibody in western blot analysis. **e** Western blot showing the significant upregulation of TPM3P9 in ccRCC cell lines compared to human kidney HK-2 cells. The black arrow represents the destination blots. **f** Western blot showing significant upregulation of TPM3P9 in ccRCC tissues compared with adjacent non-tumorous tissues. The statistical plot demonstrates the high expression of TPM3P9 in renal cancer tissues. *n* = 16. **g** The degradation half-life of TPM3P9 was detected in ACHN cells treated with cycloheximide. Data from three biological replicates are shown as mean ± SEM. **h** Immunofluorescence staining assay demonstrates the cytosolic and nuclear distribution of TPM3P9 in ccRCC cells with a TPM3P9-specific antibody. Nuclei were stained with DAPI (blue), and TPM3P9 was stained with green. Scale bar, 5 μm. **i** The nuclear-cytoplasmic fractionation experiment confirms the expression of TPM3P9 in both the cytoplasmic and nuclear compartments. **j** The expression of TPM3P9 RNA (left) or protein levels (right) in paired ccRCC and adjacent non-tumor tissues in TCGA and CPTAC cohorts, respectively. **p < 0.01; ***p < 0.001. **k** Representative immunohistochemistry staining of TPM3P9 in ccRCC with high or low expression. Scale bar,100 μm. **l**, **m** Kaplan–Meier survival analyses indicated that ccRCC patients with high TPM3P9 expression had shorter overall survival (**l**, *p* < 0.05, log-rank test) and relapse-free survival (**m**, *p* < 0.01, log-rank test)

Given the potential roles of LEPs in kidney cancer, we conducted an in-depth screening for microproteins involved in the progression of clear cell renal cell carcinoma (ccRCC), as we previously reported.^7^ We conducted nascent polypeptide-associated complex sequencing on renal tubular epithelial cells (HK-2) and renal cancer cell lines (ACHN and 786-O) to obtain noncoding RNAs bound to ribosomes to construct a database of potential LEPs (Supplementary Fig. 4a). To identify functionally relevant novel proteins in renal cancer, we performed high-throughput liquid chromatography-tandem mass spectrometry (LC-MS/MS) analysis on proteins from four renal cancer tissue samples and their adjacent non-cancerous tissues (Supplementary Fig. 4b). Seven novel proteins encoded by noncoding RNAs were identified. The heatmap illustrated the expression levels of these proteins across the four renal cancer tissues and adjacent normal tissues (Supplementary Fig. 4c). To further investigate the biological functions of the dysregulated LEPs in ccRCC, we used siRNAs to evaluate the effects of these LEPs on cell proliferation. The results showed that TPM3P9 depletion significantly inhibited the proliferation of ACHN cells (Supplementary Fig. 4d). In the SYSUCC cohort, TPM3P9 was ubiquitously expressed across nearly all types of cancer (Supplementary Fig. 4e, f). Specifically, TPM3P9 expression is higher in kidney, breast, colon, head and neck, uterine, and liver cancers compared to the corresponding adjacent normal tissues, whereas TPM3P9 expression in lung cancer tissues was lower than that in non-cancerous tissue (Fig. 2a).

TPM3P9, comprising 92 amino acids translated from two exons, is encoded by the NR_003148.3 transcript on chromosome 19 (Fig. 2b). Furthermore, we substantiated the existence of TPM3P9 protein through various experimental approaches. Shotgun mass spectrometry was used to reveal the unique peptides of TPM3P9 by comparing the peptide sequences with the reviewed human protein database downloaded from UniProt (Swiss-Prot, https://www.uniprot.org/), which contains a total of 20,435 reviewed human proteins. Through sequence alignment, we identified unique peptides specific to TPM3P9 (Fig. 2c and Supplementary Fig. 5a, b). A specific antibody for TPM3P9 was generated and used to detect endogenous and exogenous TPM3P9 protein in ccRCC cells (Fig. 2d). Western blot analyses revealed significant upregulation of TPM3P9 in ccRCC cell lines (Fig. 2e) and clinical samples (Fig. 2f). Treatment with cycloheximide revealed that the half-life of the TPM3P9 protein was ~10 h in ACHN cells (Fig. 2g). Immunofluorescence staining demonstrated the cytosolic and nuclear localization of TPM3P9 in ccRCC cells (Fig. 2h). Furthermore, we validated the expression of TPM3P9 in both the cytoplasmic and nuclear compartments through a nuclear-cytoplasmic fractionation experiment (Fig. 2i). Analysis of TCGA and CPTAC data showed that both the mRNA and protein expression levels of TPM3P9 were higher in ccRCC tissues than in the corresponding adjacent non-tumor tissues. (Fig. 2j). Immunohistochemistry was used to evaluate the expression of TPM3P9 in 385 ccRCC patients (Fig. 2k and Supplementary Table 1), showing higher expression of TPM3P9 in ccRCC tissues. Prognostic analyses indicated that ccRCC patients with high TPM3P9 expression had shorter overall survival (OS) and relapse-free survival (RFS) (Fig. 2l, m). Overall, these findings indicate that lncRNA-TPM3P9 is able to be translated into the new microprotein TPM3P9, which is abnormally upregulated in ccRCC and associated with poor prognosis.

### TPM3P9 exerts oncogenic activity in ccRCC

**Fig. 3:**
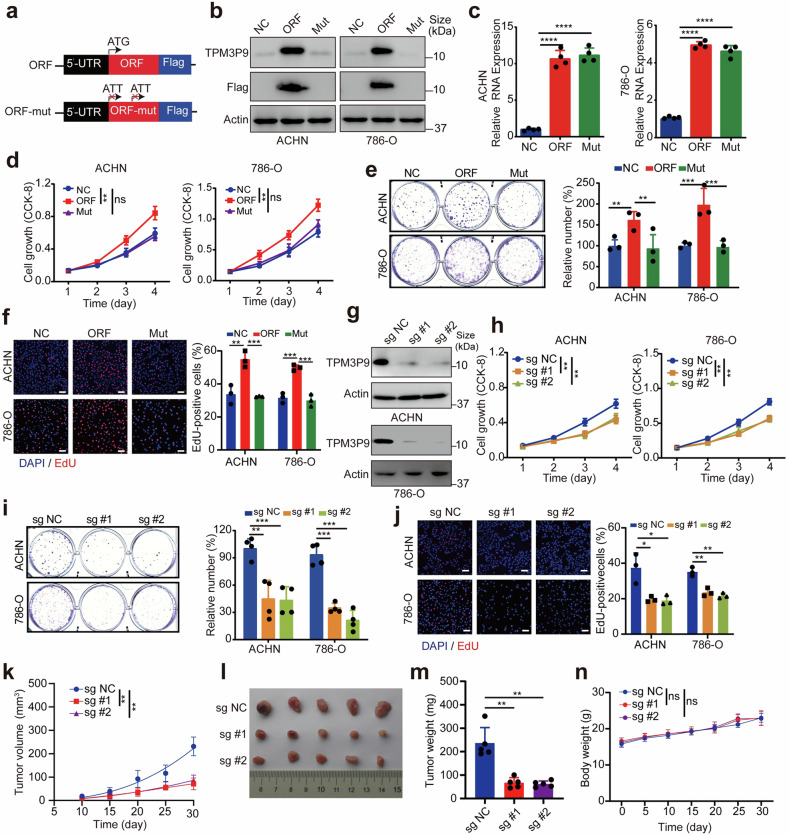
The novel microprotein TPM3P9 promotes ccRCC proliferation. **a** Two plasmids encoding TPM3P9-Flag (TPM3P9) and TPM3P9-mut-Flag (Mut) were constructed. To generate the mutant, the translation initiation codon ATG was mutated to ATT to abolish protein translation. **b** Western blot analysis using anti-TPM3P9 and anti-Flag antibodies showed that the TPM3P9 protein was overexpressed only in ccRCC cells with the wild-type ORF. **c** TPM3P9 mRNA expression was upregulated in ccRCC cells transfected with either the wild-type or mutant TPM3P9 ORF. ****p < 0.0001. **d** CCK-8 assays were performed to test the growth ability of both ccRCC cell lines transfected with the indicated constructs. **p < 0.01; ns, nonsignificant. **e** Colony formation assays were performed to assess the colony formation ability of both ccRCC cell lines transfected with the indicated constructs. **p < 0.01; ***p < 0.001. **f** EdU assays were performed to evaluate the proliferation ability of both ccRCC cells transfected with the indicated constructs. **p < 0.01; ***p < 0.001. Bars, SEMs. Scale bar, 50 μm. **g** Western blot validation of TPM3P9 knockdown efficiency of the indicated sgRNAs in ccRCC cells. **h** CCK-8 assays were performed to test the growth ability of both ccRCC cell lines transfected with the indicated sgRNAs. **p < 0.01. **i** Colony formation assays were performed to test the colony formation ability of ccRCC cells transfected with the indicated sgRNAs. **p < 0.01; ***p < 0.001. Bars, SEMs. **j** EdU assays were performed to test the proliferation ability of both ccRCC cell lines transfected with the indicated sgRNAs. *p < 0.05; **p < 0.01. Bars, SEMs. Scale bar, 50 μm. **k** Mouse xenograft model showing that knockdown of TPM3P9 significantly inhibited tumor growth. **p < 0.01. **l**, **m** A mouse xenograft model was established by injection of control or TPM3P9-silenced ACHN cells, with tumor volume (**l**) and tumor weight (**m**) shown. Data from 5 mice per group are presented. **p < 0.01. **n** The body weight of the nude mice was monitored. ns, nonsignificant

We next investigated the effect of the microprotein TPM3P9 on the proliferation of ccRCC cells. We constructed a plasmid containing the ORF encoding the wild-type TPM3P9 protein as well as a plasmid containing the related ORF with mutation of the start codon ATG to ATT, which normally results in the expression of the lncRNA but not the protein (Fig. 3a). TPM3P9 mRNA expression was upregulated in cells transfected with either the wild-type or the mutant ORF, whereas the TPM3P9 protein was overexpressed only in cells transfected with the wild-type ORF (Fig. 3b, c). CCK-8 (Fig. 3d), colony formation (Fig. 3e), and EdU incorporation assays (Fig. 3f) were performed to evaluate the effects of TPM3P9 protein overexpression on cell growth. The results demonstrated that overexpression of the TPM3P9 protein but not the associated lncRNA promoted ccRCC cell proliferation. Furthermore, we conducted qRT-PCR to measure the expression levels of lncRNA-TPM3P9 in several ccRCC cell lines. The results demonstrated that in ACHN and 786-O cells, the baseline expression of lncRNA-TPM3P9 was not significantly higher than in renal tubular epithelial cells HK-2 (Supplementary Fig. 6a). This suggests that the lack of significant cellular function observed with the overexpression of mutant start codon lncRNA-TPM3P9 is not due to high baseline expression of lncRNA. On the other hand, sgRNAs were used to knock down TPM3P9 in ACHN and 786-O cells. Western blot analysis confirmed the depletion of the TPM3P9 protein (Fig. 3g). The RNA expression level of TPM3P9 was not affected by sgRNAs (Supplementary Fig. 6b). Sequencing of the DNA fragments flanking the sgRNA target sites, combined with the T7 endonuclease I assay, further demonstrated that the knockout system functioned effectively at the DNA level (Supplementary Fig. 6c, d). Silencing TPM3P9 markedly suppressed cell viability, inhibited colony formation, reduced the number of EdU-positive cells, and induced G1 arrest (Fig. 3h–j and Supplementary Fig. 6e). Strikingly, both the overexpression and knockdown of TPM3P9 in ccRCC cells had limited effects on apoptosis (Supplementary Fig. 6f, g). In vivo models were used to examine the impact of TPM3P9 depletion on tumor growth. Tumor growth was significantly inhibited by knockout of TPM3P9 (Fig. 3k). Compared to control ccRCC cells, ccRCC cells containing sgRNAs specific for TPM3P9 generated xenograft tumors with a significantly smaller size and lower weight (Fig. 3l, m). No significant difference was observed in body weight among the mice (Fig. 3n). Collectively, these results indicate that the microprotein TPM3P9 promotes ccRCC progression in vivo and in vitro.

### TPM3P9 regulates the splicing of the oncogenic RNA TCF7L2

**Fig. 4:**
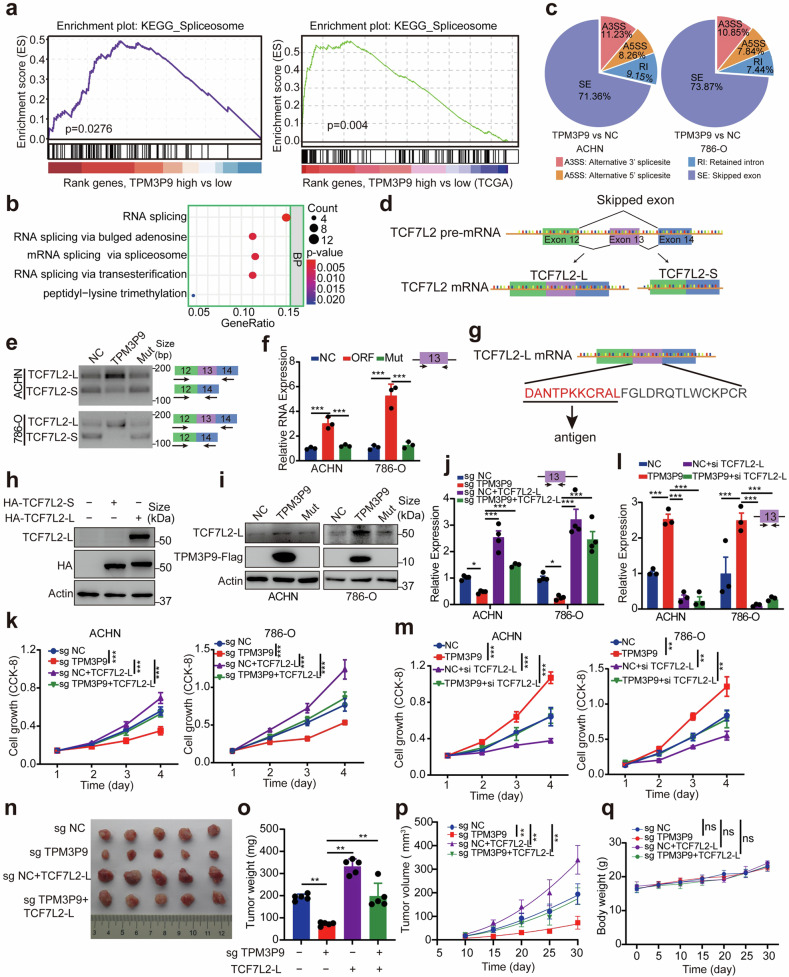
The microprotein TPM3P9 regulates the oncogenic RNA splicing of TCF7L2. **a** GSEA of ccRCC proteomic data and TCGA data showing that TPM3P9 is involved in the spliceosome pathway. **b** Enrichment analysis suggested that the proteins co-expressed with TPM3P9 were related to RNA splicing. **c** Pie chart based on RNA sequencing showing the distribution of alternative splicing changes following TPM3P9 overexpression in ACHN and 786-O cells. **d** Schematic representation of TCF7L2 alternative splicing and its splice variants. **e** RT-PCR validation of the increased expression of the TCF7L2-L variant in cells overexpressing the TPM3P9 protein. **f** The results of qRT-PCR using a specific primer pair confirmed the increase in the expression of the TCF7L2-L variant upon overexpression of the TPM3P9 protein.***p < 0.001.  **g** Preparation of an antibody specific to the TCF7L2-L variant. **h** Western blot validation of the successful preparation of the anti-TCF7L2-L antibody and exogenous overexpression of the TCF7L2-L and TCF7L2-S variants. The HA-tag was fused with TCF7L2-L or TCF7L2-S. **i** Western blot showing the upregulation of the TCF7L2-L variant in cells ectopically expressing TPM3P9-Flag (TPM3P9) but not in those ectopically expressing TPM3P9-mut-Flag (Mut). **j**, **k** Knockdown of TCF7L2-L inhibited the ability of TPM3P9 to promote ccRCC cell proliferation. The expression of TCF7L2-L was measured by qRT-PCR (**j**), and the cell growth ability was confirmed by a CCK-8 assay (**k**). *p < 0.05; ***p < 0.001. Bars, SEMs. **l**, **m** Overexpression of TCF7L2-L reversed the inhibitory effect of TPM3P9 silencing on ccRCC cell growth. The expression of TCF7L2-L was measured by qRT-PCR (**l**), and the cell growth ability was confirmed by a CCK-8 assay (**m**). **p < 0.01; ***p < 0.001. Bars, SEMs. **n**–**p** Mouse xenograft model showing that overexpression of TCF7L2-L reversed TPM3P9 silencing-mediated inhibition of proliferation in ACHN cells. Tumor volume (**n**), tumor weight (**o**), and the increase in tumor volume over time (**p**) in nude mice. **p < 0.01; ns, nonsignificant. Bars, SD. **q** The body weight of the nude mice was monitored. ns, nonsignificant

The molecular mechanism through which TPM3P9 promotes ccRCC was subsequently explored. Gene set enrichment analysis (GSEA) of the ccRCC proteomic data and TCGA data based on the TPM3P9 expression revealed that TPM3P9 is involved in spliceosome signaling (Fig. 4a). Co-expression network analysis revealed that pathways related to RNA splicing were enriched in proteins co-expressed with TPM3P9 (Fig. 4b and Supplementary Fig. 7a). Thus, we performed RNA sequencing analysis of TPM3P9-overexpressing ACHN and 786-O cells to examine TPM3P9-mediated alternative RNA splicing (Fig. 4c and Supplementary Fig. 7b). The results indicated that exon skipping was the main RNA splicing event associated with TPM3P9. Among the candidate genes, *TCF7L2* exhibited the most pronounced changes in exon retention. Specifically, retention of exon 13 in TCF7L2 mRNA was increased by ectopic expression of TPM3P9, leading to increased expression of the long splice variant TCF7L2-L (Fig. 4d and Supplementary Fig. 7c). The RT-PCR results showed that overexpression of the TPM3P9 protein, but not the corresponding mRNA, markedly induced the expression of TCF7L2-L and reduced the expression of TCF7L2-S in ccRCC cells (Fig. 4e). This was further confirmed by qRT-PCR using primers specific for TCF7L2-L (Fig. 4f).

Next, we examined the expression levels of TPM3P9 protein and TCF7L2-L mRNA in 12 ccRCC tissue samples. High TPM3P9 expression was tremendously associated with higher TCF7L2-L mRNA levels in clinical samples (Supplementary Fig. 7d). Furthermore, plasmids encoding TCF7L2-L and TCF7L2-S were constructed (Supplementary Fig. 7e) and an antibody specifically recognizing TCF7L2-L was generated (Fig. 4g). Both plasmids and the antibody were validated by western blot analysis (Fig. 4h). Ectopic expression of TPM3P9 in ccRCC cells upregulated TCF7L2-L protein expression (Fig. 4i). Silencing TPM3P9 significantly decreased TCF7L2-L mRNA expression (Fig. 4j).

To determine whether TPM3P9 promotes cell proliferation through TCF7L2-L, TCF7L2-L was re-expressed in ccRCC cells following TPM3P9 depletion (Fig. 4j). Restoration of TCF7L2-L expression reversed the inhibitory effect of TPM3P9 depletion on ccRCC cell growth (Fig. 4k). On the other hand, TCF7L2-L was knocked down in cells with TPM3P9 overexpressed (Fig. 4l). Silencing TCF7L2-L impeded TPM3P9-mediated cell proliferation (Fig. 4m). TCF7L2-L overexpression facilitated but TCF7L2-L knockdown inhibited ccRCC cell proliferation in vitro (Fig. 4k–m). Furthermore, results from the xenograft model demonstrated that TCF7L2-L overexpression increased xenograft growth and was able to abolish the suppression of tumor growth resulting from TPM3P9 knockout (Fig. 4n–p). The body weights of the nude mice were monitored, and no significant difference was found (Fig. 4q). Taken together, these findings indicate that the microprotein TPM3P9 modulates the splicing of TCF7L2 RNA to generate the oncogenic splice variant TCF7L2-L in ccRCC cells.

### TPM3P9 suppresses TCF7L2 exon skipping *via* interaction with the RRM1 domain of the splicing factor RBM4

**Fig. 5:**
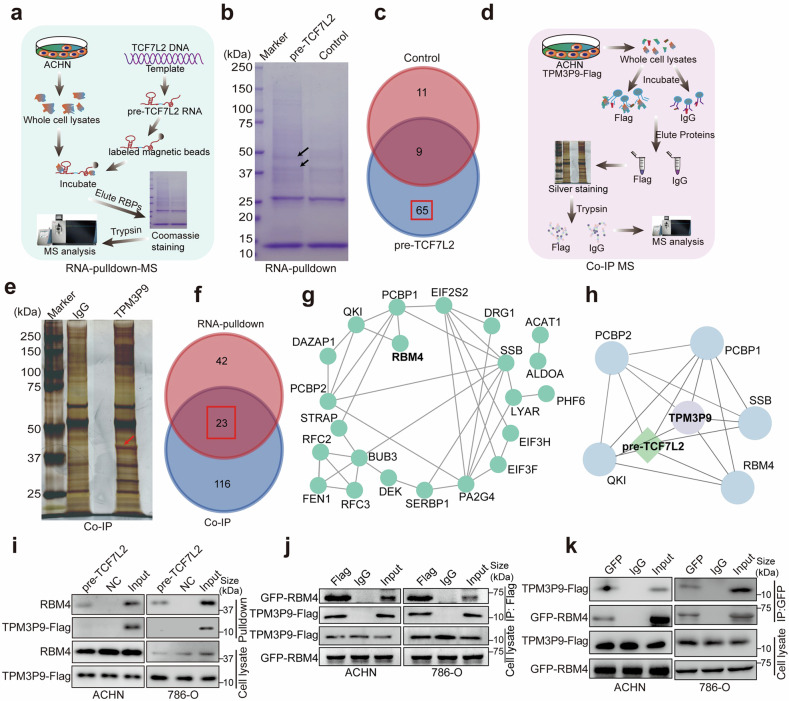
The microprotein TPM3P9 binds to RBM4 and suppresses TCF7L2 exon skipping. **a** Diagram showing the workflow of RNA pulldown combined with high-resolution mass spectrometry to identify proteins binding to TCF7L2 pre-mRNA. The diagram was edited using Adobe Illustrator. **b** Coomassie blue staining showing TCF7L2 pre-mRNA pulled down with RNA probes. The black arrows represent the lanes with significant differences. **c** Venn diagram showing a total of 65 proteins potentially interacting with TCF7L2 pre-mRNA. **d** Diagram showing the workflow of Co-IP combined with high-resolution mass spectrometry to identify TPM3P9-interacting proteins. The diagram was edited using Adobe Illustrator. **e** Silver staining showing proteins specifically bind to TPM3P9; specific bands are highlighted with red arrows. **f** Venn diagram showing 23 proteins potentially interacting with both TPM3P9 and the TCF7L2 pre-mRNA. **g** A PPI network of these proteins potentially interacting with both TPM3P9 and the TCF7L2 pre-mRNA was constructed using Cytoscape software. **h** The PPI network of the five interaction partners closely associated with TPM3P9 and the TCF7L2 pre-mRNA. **i** RNA pulldown with the TCF7L2 pre-mRNA probe was performed to validate the interaction between TCF7L2 pre-mRNA and RBM4. **j** Co-IP assays using an anti-Flag antibody were performed to detect the interaction between TPM3P9 and RBM4 in ACHN and 786-O cells. **k** Co-IP assays using an anti-GFP antibody were performed to detect the interaction between TPM3P9 and RBM4 in ACHN and 786-O cells expressing TPM3P9-Flag

We next investigated the mechanism by which TPM3P9 regulates TCF7L2 alternative splicing. Since no RNA-binding motif was found in TPM3P9, we hypothesized that TPM3P9 mediates RNA alternative splicing *via* interaction with splicing factors. RNA pulldown combined with high-resolution mass spectrometry was performed to identify proteins that bind to TCF7L2 pre-mRNA (Fig. 5a). Coomassie Brilliant Blue staining was used to visualize the proteins pulled down by the RNA probes (Fig. 5b). A total of 65 proteins were uniquely identified in the TCF7L2 group (Fig. 5c and Supplementary Table 2). Gene Ontology analysis revealed significant enrichment of the term RNA splicing (Supplementary Fig. 8a). Simultaneously, we conducted immunoprecipitation-mass spectrometry (IP-MS) to identify TPM3P9-interacting proteins (Fig. 5d). Silver staining was performed to detect proteins that specifically bind to TPM3P9 (Fig. 5e). MS analysis identified 139 proteins across four independent biological replicates of samples precipitated using an antibody specific for Flag (Supplementary Fig. 8b–d and Supplementary Table 3). Gene Ontology analysis revealed significant enrichment of the term RNA-binding pathway (Supplementary Fig. 8e). We next compared the TCF7L2 pre-mRNA-binding proteins with the TPM3P9-binding proteins and identified 23 overlapping candidates (Fig. 5f). The protein interaction network revealed the relationships among these candidates (Fig. 5g).

Additionally, we used catRAPID^23,24^ to predict RNA-binding proteins that may interact with TCF7L2 pre-mRNA (Supplementary Table 4). By intersecting these predicted proteins with the 23 candidate proteins, we found five proteins with strong evidence for interactions with both TPM3P9 and TCF7L2 pre-mRNA (Fig. 5h). Among them, RBM4, an RNA-binding protein involved in the alternative splicing of numerous genes, was chosen for further investigation. We first verified the binding of RBM4 to TCF7L2 pre-mRNA by RNA pulldown experiments, demonstrating that RBM4 but not TPM3P9 was detectable in the precipitates obtained with the TCF7L2 pre-mRNA probe (Fig. 5i). Subsequently co-immunoprecipitation assays and confocal microscopy confirmed the interaction between RBM4 and TPM3P9 in the nucleus of ccRCC cells (Fig. 5j, k and Supplementary Fig. 8f).

**Fig. 6:**
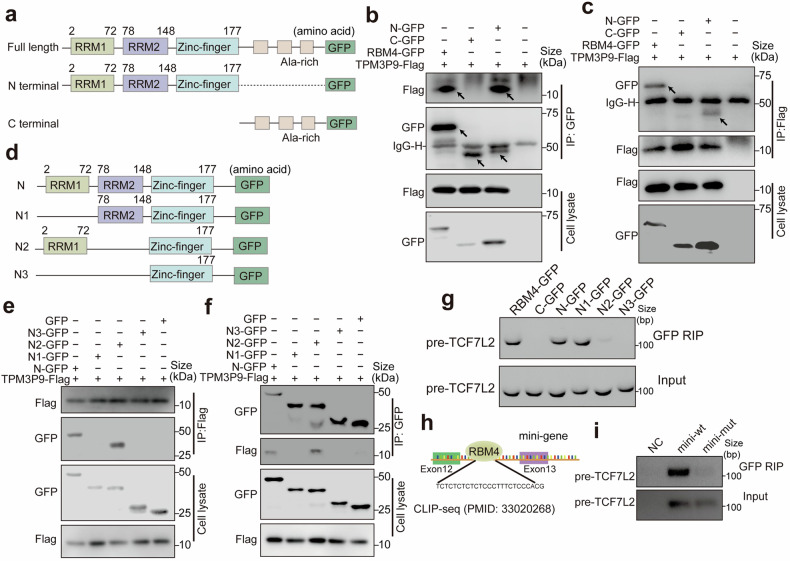
The exploration of the binding region of TPM3P9 and RBM4. **a** Diagram of plasmids encoding GFP-tagged full-length RBM4 (RBM4-GFP) and GFP-tagged RBM4 truncations (RBM4-N-GFP and RBM4-C-GFP). The diagram was edited using Adobe Illustrator. **b** The indicated GFP-tagged wild-type RBM4 and mutant RBM4 plasmids as well as the TPM3P9-Flag plasmid were transfected into HEK293T cells, and co-immunoprecipitation with an antibody specific to GFP revealed that RBM4-GFP and RBM4-N-GFP, but not RBM4-C-GFP, were capable of binding to TPM3P9. The black arrows represent the destination blots. **c** The indicated GFP-tagged wild-type RBM4 and mutant RBM4 plasmids as well as the TPM3P9-Flag plasmid were transfected into HEK293T cells, and coimmunoprecipitation with an antibody specific for Flag revealed that RBM4-GFP and RBM4-N-GFP but not RBM4-C-GFP were capable of binding to TPM3P9. The black arrows represent the destination blots. **d** Diagram of plasmids encoding the RBM4 N-terminal truncations lacking RRM1 (N1-GFP), RRM2 (N2-GFP), or both RRM1 and RRM2 (N3-GFP). The diagram was edited using Adobe Illustrator. **e**, **f** The indicated GFP-tagged N-terminal domain deletion mutants of RBM4 were co-transfected with TPM3P9-Flag into HEK293T cells, and co-immunoprecipitation was performed using an antibody specific for Flag (**e**) or GFP (**f**); the results showed that both N1-GFP and N3-GFP, without the RRM1 motif, could not bind to TPM3P9. **g** RNA immunoprecipitation (RIP) showing the binding ability of different RBM4 truncations to TCF7L2 pre-mRNA. **h** Crosslinking immunoprecipitation coupled with high-throughput sequencing (CLIP-seq) analysis of RBM4 revealed that RBM4 specifically crosslinked to an intron upstream of exon 13 in TCF7L2 pre-mRNA. **i** The minigene assay and RIP assay using a binding sequence mutant with a mutation in the intron upstream of exon 13 confirmed that RBM4 bound only to the wild-type TCF7L2 pre-mRNA

RBM4 primarily consists of an N-terminal RNA recognition motif (RRM) domain, responsible for RNA binding, and a C-terminal domain involved in protein-protein interactions.^25^ We constructed truncations to determine whether the RNA-binding domain of RBM4 is essential for its interaction with TPM3P9. We constructed plasmids encoding GFP-tagged full-length (RBM4-GFP) or GFP-tagged RBM4 truncation (RBM4-N-GFP, and RBM4-C-GFP) (Fig. 6a). These plasmids were co-transfected with the TPM3P9-Flag plasmid into ccRCC cells. Co-immunoprecipitation experiments using antibodies specific for GFP or Flag revealed that RBM4-GFP and RBM4-N-GFP but not RBM4-C-GFP were capable of binding to TPM3P9 (Fig. 6b, c). Given that the N-terminus of RBM4 contains two RNA-binding motifs, RRM1 and RRM2, we further constructed plasmids expressing N-terminal truncations lacking RRM1 (N1-GFP), RRM2 (N2-GFP), or both RRM1 and RRM2 (N3-GFP) (Fig. 6d). As depicted in Fig. 6e, f, both N1-GFP and N3-GFP lost the ability to bind to TPM3P9, indicating the crucial role of RRM1 domain in the RBM4-TPM3P9 interaction.

The binding of RBM4 to TCF7L2 pre-mRNA was further characterized using RNA immunoprecipitation (RIP). We found that RBM4-ΔRRM1 was able to bind to TCF7L2 pre-mRNA. However, the ability to bind TCF7L2 pre-mRNA was lost when RRM2 or both RRM1 and RRM2 domains were absent (Fig. 6g). This indicated that the RRM2 domain of RBM4 was required for its interaction with TCF7L2 pre-mRNA. Analysis of crosslinking immunoprecipitation-high-throughput-sequencing (CLIP-seq) data for RBM4^26^ revealed the specific RBM4-binding site in TCF7L2 pre-mRNA (Fig. 6h), revealing that RBM4 was cross-linked to an intron upstream of exon 13. The results of minigene and RIP assays using a mutated binding sequence in the intron demonstrated that RBM4 bound exclusively to the wild-type TCF7L2 pre-mRNA (Fig. 6i). These findings indicated that TPM3P9 modulates RNA splicing *via* its interaction with RBM4 in ccRCC cells.

### TPM3P9 inhibits RBM4-mediated TCF7L2 RNA splicing

**Fig. 7:**
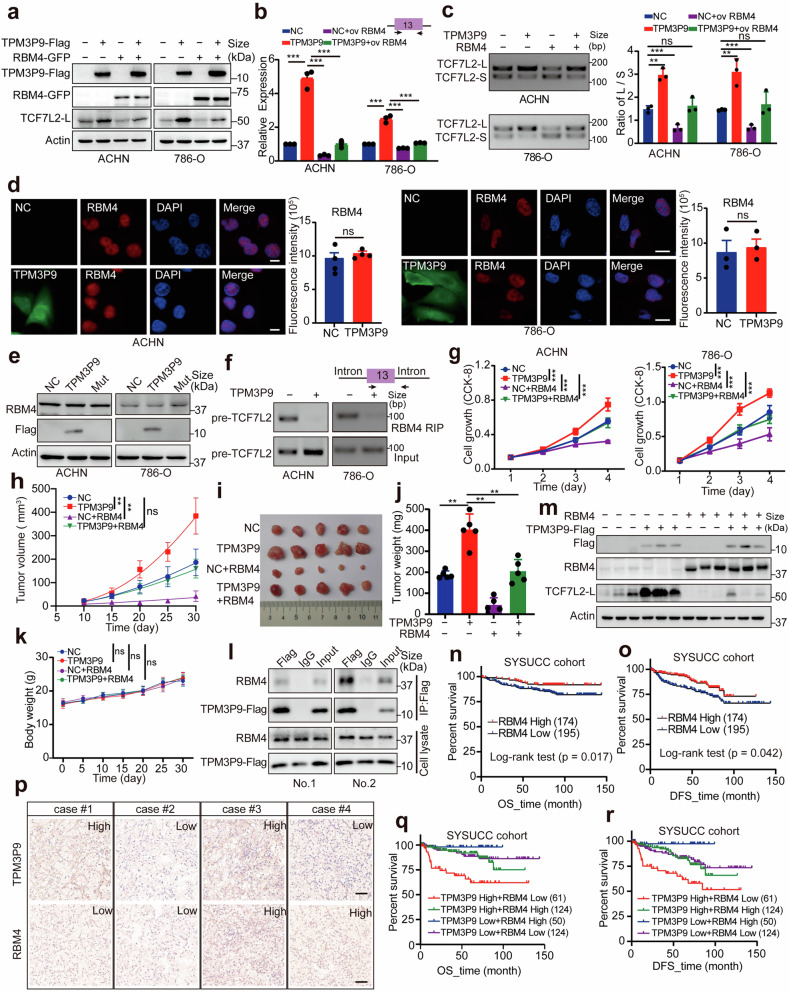
The microprotein TPM3P9 inhibits RBM4-mediated TCF7L2 RNA splicing. **a** Western blot results showing that overexpression of RBM4 dramatically abrogated the TPM3P9-mediated increase in TCF7L2-L protein expression. **b** qRT-PCR results showing that RBM4 dramatically abrogated the TPM3P9-mediated increase in TCF7L2-L mRNA expression. ***p < 0.001. **c** RT-PCR results showing that the increase in the TCF7L2-L/TCF7L2-S ratio induced by TPM3P9 overexpression was reversed to the control level when RBM4 was overexpressed. **p < 0.01; ***p < 0.001; ns, nonsignificant. **d** Immunofluorescence staining showing that TPM3P9 overexpression did not affect the cellular localization or expression of the RBM4 protein. Nuclei were stained with DAPI (blue), TPM3P9 (green), and RBM4 (red). The staining intensity of RBM4 (red) was quantified. Unpaired two-tailed Student’s *t*-test; ns, nonsignificant. Bars, SEMs; scale bars, 10 μm (left) and 20 μm (right). **e** Western blot results showing that TPM3P9 overexpression did not affect the expression of the RBM4 protein in ccRCC cells. **f** RIP assay results showing that TPM3P9 overexpression weakened the binding ability of RBM4 to TCF7L2 pre-mRNA in ccRCC cells. **g** Overexpression of RBM4 restored the TPM3P9-mediated promotion of ccRCC cell growth. ***p < 0.001. **h** Mouse xenograft model showing that overexpression of RBM4 significantly inhibited tumor growth promoted by TPM3P9 overexpression. **p < 0.01; ns, nonsignificant. **i**, **j** Mouse xenograft model established with ACHN cells. Tumor growth (**i**), and tumor weight (**j**) are shown. Data are shown as the mean ± SD for 5 mice per group. Unpaired two-tailed Student’s *t*-test; **p < 0.01. **k** The body weight of the nude mice was monitored. ns, nonsignificant. **l** Co-IP assay results verifying the interaction between RBM4 and TPM3P9 in in vivo tumor samples. **m** Western blot results showing that RBM4 overexpression abolished the upregulation of TCF7L2-L mediated by TPM3P9 in in vivo tumor samples. **n** Kaplan–Meier survival analysis results showing that high RBM4 expression was associated with favorable overall survival in ccRCC patients in the SYSUCC cohort (*p* < 0.05, log-rank test). **o** Kaplan–Meier survival analysis showing that high RBM4 expression was associated with favorable disease-free survival in ccRCC patients in the SYSUCC cohort (*p* < 0.05, log-rank test). **p** Immunohistochemical data revealing the correlation between TPM3P9 and RBM4 expression in ccRCC patients. Scale bars, 50 μm. **q** Kaplan–Meier analysis of overall survival revealed that in the SYSUCC cohort, ccRCC patients with both high TPM3P9 and low RBM4 expression had the worst prognosis, while patients with both low TPM3P9 and high RBM4 expression had the best prognosis (*p* < 0.05, log-rank test). **r** Kaplan–Meier analysis of disease-free survival showed that in the SYSUCC cohort, ccRCC patients with both high TPM3P9 and low RBM4 expression had the worst prognosis, while patients with both low TPM3P9 and high RBM4 expression had the best prognosis (*p* < 0.05, log-rank test)

We next determined the role of RBM4 in the splicing of TCF7L2 RNA. Overexpression of RBM4 in ccRCC cells increased the expression of TCF7L2-S but decreased the expression of TCF7L2-L (Supplementary Fig. 9a, b). Furthermore, exogenous expression of RBM4 in ccRCC cells overexpressing TPM3P9 attenuated the upregulation of TCF7L2-L (Fig. 7a, b). The increase in the TCF7L2-L/TCF7L2-S ratio mediated by TPM3P9 overexpression was rescued by overexpression of RBM4 (Fig. 7c). To explore whether TPM3P9 is able to modulate the expression of RBM4, confocal microscopy and western blot analysis were performed. The results showed that TPM3P9 overexpression did not affect the cellular localization or expression level of the RBM4 protein in ccRCC cells (Fig. 7d, e). Considering our previous finding that RBM4 interacts with TPM3P9 and the TCF7L2 pre-mRNA through a similar domain, we next examined whether TPM3P9 modulates the binding of RBM4 to TCF7L2 pre-mRNA. RIP assays showed that RBM4 lost its ability to pull down TCF7L2 pre-mRNA in the presence of TPM3P9 overexpression (Fig. 7f). These findings indicate that TPM3P9 binds to the RRM domain of RBM4 to block RBM4-mediated RNA splicing of TCF7L2.

We further explored the impact of RBM4 on the ability of TPM3P9 to promote cell growth. Our in vitro and in vivo experiments demonstrated that overexpression of TPM3P9 in ccRCC cells increased cell viability and promoted tumor growth (Fig. 7g–j). Conversely, the ability of TPM3P9 to promote ccRCC cell proliferation and tumor growth was markedly suppressed by overexpression of RBM4 (Fig. 7g–j). The body weights of the nude mice remained unchanged (Fig. 7k). The results of Co-IP and western blot analysis of tumor samples from the in vivo model confirmed the interaction between TPM3P9 and RBM4 (Fig. 7l), and showed that TPM3P9 upregulated the expression of TCF7L2-L, an effect that was abolished by RBM4 overexpression (Fig. 7m). Collectively, these data suggest that TPM3P9 exerts its pro-tumor effect by modulating RBM4-mediated RNA splicing.

The expression of RBM4 in ccRCC tissues and its clinical significance were subsequently examined. Analysis of the network database revealed that RBM4 expression was significantly lower in ccRCC tissues than in non-tumor tissues (Supplementary Fig. 9c). A low RBM4 expression level was significantly correlated with a high clinical stage (Supplementary Fig. 9d) and more lymph node metastasis (Supplementary Fig. 9e). High RBM4 expression was associated with favorable overall and disease-free survival in both the TCGA and SYSUCC cohorts (Fig. 7n, o and Supplementary Fig. 9f). Immunohistochemical analysis revealed no significant correlation between TPM3P9 and RBM4 expression in patients with ccRCC (Fig. 7p). However, patients with both high TPM3P9 and low RBM4 expression had the worst prognosis, while patients with both low TPM3P9 and high RBM4 expression had the best prognosis (Fig. 7q, r).

### TCF7L2-L transcriptionally upregulates RELB to activate NF-κB signaling

**Fig. 8:**
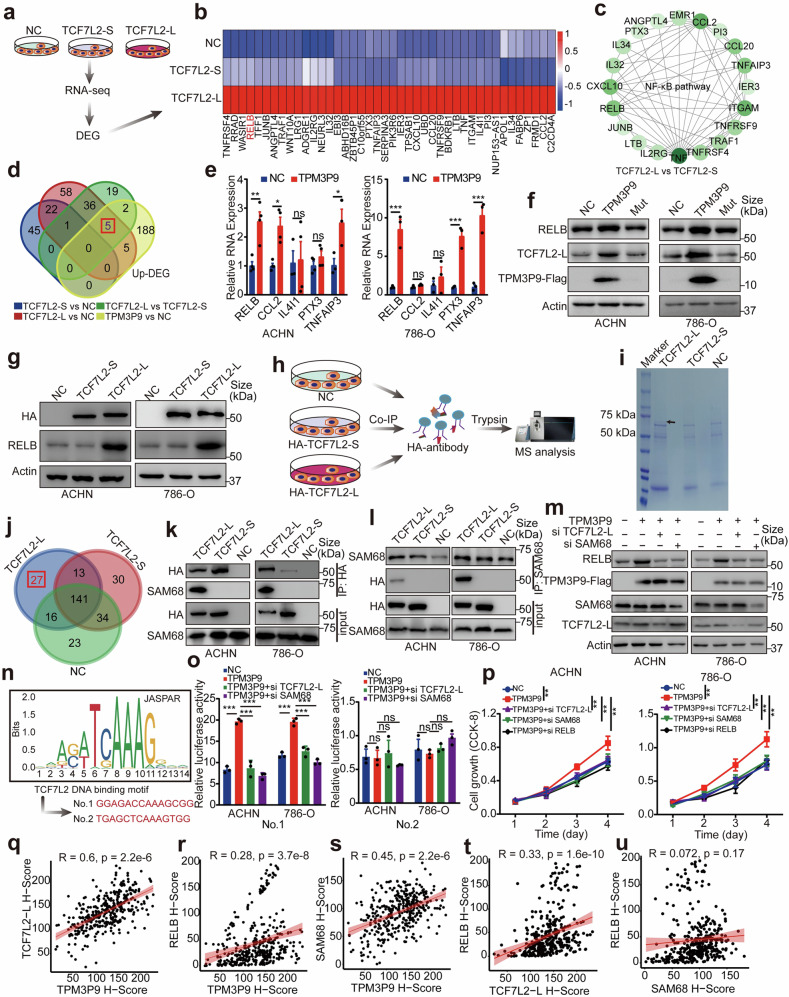
TCF7L2-L transcriptionally upregulates RELB to activate NF-κB signaling. **a** Diagram showing the process for RNA-seq analysis of ccRCC cells with control plasmid, TCF7L2-L variant, or TCF7L2-S variant overexpression. **b** Heatmap showing the genes upregulated in cells transfected with TCF7L2-L but with unchanged expression in cells transfected with TCF7L2-S compared to control cells. **c** Comparative analysis of the TCF7L2-S and TCF7L2-L groups revealing that the NF-κB pathway was the predominant pathway enriched in the differentially expressed proteins. **d** Five essential upregulated genes (RELB, CCL2, IL4I1, PTX3, and TNFAIP3) were identified by overlapping the genes upregulated by overexpression of TCF7L2-L with those upregulated by overexpression of TPM3P9. **e** qRT-PCR was performed to validate the upregulation of RELB and TNFAIP3 mRNA expression after the overexpression of TPM3P9 in ACHN and 786-O cells. *p < 0.05; **p < 0.01; ***p < 0.001; ns, nonsignificant. **f** Western blot analysis confirmed that the overexpression of the TPM3P9 protein, but not the corresponding lncRNA, upregulated RELB protein expression in ccRCC cells. **g** Western blot analysis confirmed that ectopic expression of the TCF7L2-L variant but not the TCF7L2-S variant noticeably induced the expression of RELB in ccRCC cells. **h** Co-IP using an anti-HA antibody followed by MS was performed to identify the coregulatory factors of TCF7L2-L. The diagram was edited using Adobe Illustrator. **i** Coomassie blue staining showing the proteins that specifically bind to TCF7L2-L; specific bands are highlighted with black arrows. **j** The Venn diagram shows that 27 proteins that bound specifically to TCF7L2-L but not to TCF7L2-S. **k** Co-IP assays were performed using an anti-HA antibody to verify the interaction between the HA-TCF7L2-L variant and SAM68 in ACHN and 786-O cells. The HA-TCF7L2-S variant and the empty vector were used as controls. **l** Co-IP assays were performed using an anti-SAM68 antibody to detect the interaction between TCF7L2-L and SAM68 in ccRCC cells. **m** Western blot analysis confirmed that silencing TCF7L2-L or SAM68 noticeably attenuated the induction of RELB expression in ccRCC cells overexpressing TPM3P9. **n** The predicted sequence motifs for TCF7L2 binding DNA. Two potential sequences in the RELB promoter were predicted to bind to TCF7L2. **o** Dual luciferase reporter assays revealed that the activity of motif No.1 but not that of motif No.2 was increased by TPM3P9 overexpression, and this increase was strongly attenuated by treatment with siRNA targeting TCF7L2-L or SAM68. ***p < 0.001; ns, nonsignificant. **p** CCK-8 assays were performed to test the growth ability of ccRCC cells transfected with the indicated constructs. **p < 0.01. **q**–**u** Immunohistochemical and correlation analyses were performed in the SYSUCC cohort, comprising 385 clinical samples, to analyze the expression correlations of TPM3P9 with TCF7L2-L (**q**), RELB with TPM3P9 (**r**), SAM68 with TPM3P9 (**s**), RELB with TCF7L2-L (**t**), and RELB with SAM68 (**u**)

Transcriptomic analysis was also conducted to explore the mechanism by which TCF7L2-L promotes the cell proliferation in ccRCC (Fig. 8a). The results revealed genes upregulated in cells transfected with TCF7L2-L but unchanged in cells transfected with TCF7L2-S compared to the control cells (Fig. 8b). Comparative analyses of the TCF7L2-S and TCF7L2-L transfection groups revealed that the NF-κB pathway was the predominant pathway enriched in the differentially expressed genes (Fig. 8c). Thus, we further determined the intersection between the genes upregulated by the overexpression of TCF7L2-L and those upregulated by overexpression of TPM3P9. Five essential genes including RELB, CCL2, IL4I1, PTX3, and TNFAIP3 were found (Fig. 8d), and the regulation of these five genes by TPM3P9 was next examined. qRT-PCR revealed that the mRNA expression levels of RELB and TNFAIP3 were increased in both ACHN and 786-O cells transfected with TPM3P9 (Fig. 8e). Due to its essential role in NF-κB signaling, RELB was selected for further investigation. Western blot analysis confirmed that overexpression of the TPM3P9 protein in renal cancer cells upregulated RELB expression. In contrast, overexpression of the mutant lncRNA-TPM3P9 (Mut), which could not encode the protein, did not affect RELB expression (Fig. 8f). Furthermore, we examined RELB expression in RCC cells with TPM3P9 knockdown. The results indicated that TPM3P9 silencing inhibited RELB expression (Supplementary Fig. 10a). Importantly, ectopic expression of TCF7L2-L markedly induced the expression of RELB, whereas ectopic expression of the TCF7L2-S variant did not (Fig. 8g).

We next determined how TCF7L2-L transcriptionally upregulates RELB. IP-MS was performed using an anti-HA antibody to identify the co-regulatory factors of TCF7L2-L (Fig. 8h). Coomassie blue staining showed the protein band specific to cells overexpressing HA-TCF7L2-L (Fig. 8i). The MS results revealed 27 proteins that specifically bound to TCF7L2-L and not to TCF7L2-S (Fig. 8j and Supplementary Table 5). SAM68, an oncogenic protein involved in the NF-κB pathway, was selected for further study. Co-IP experiments verified that TCF7L2-L but not TCF7L2-S interacted with SAM68 (Fig. 8k, l). To determine whether the interaction between TCF7L2-L and SAM68 affects TPM3P9-mediated RELB upregulation, TCF7L2-L or SAM68 was knocked down in ccRCC cells expressing TPM3P9. The experimental results showed that silencing of either TCF7L2-L or SAM68 noticeably attenuated the induction of RELB expression in cells overexpression TPM3P9 (Fig. 8m). DNA binding motif analysis of the RELB promoter indicated two potential binding sites for TCF7L2-L (Fig. 8n). Dual luciferase reporter assays revealed that the activity of motif No.1 but not that of motif No.2 was increased by TPM3P9, and this increase was strongly attenuated by treatment with siRNA targeting TCF7L2-L or SAM68 (Fig. 8o). In addition, the CCK-8 assay demonstrated that the TPM3P9-mediated proliferative phenotype of ccRCC cells was inhibited by the silencing of TCF7L2-L, SAM68, or RELB (Fig. 8p). In the 385 clinical samples from the SYSUCC cohort, TPM3P9 protein expression was significantly associated with the expression of TCF7L2-L, RELB and SAM68, while RELB expression was positively correlated with the expression of TCF7L2-L and SAM68 (Fig. 8q–u and Supplementary Fig. 10b–f). Collectively, our data suggest that TPM3P9 upregulates RELB expression to trigger NF-κB signaling *via* the TCF7L2-L/SAM68 complex in ccRCC cells.

## Discussion

**Fig. 9:**
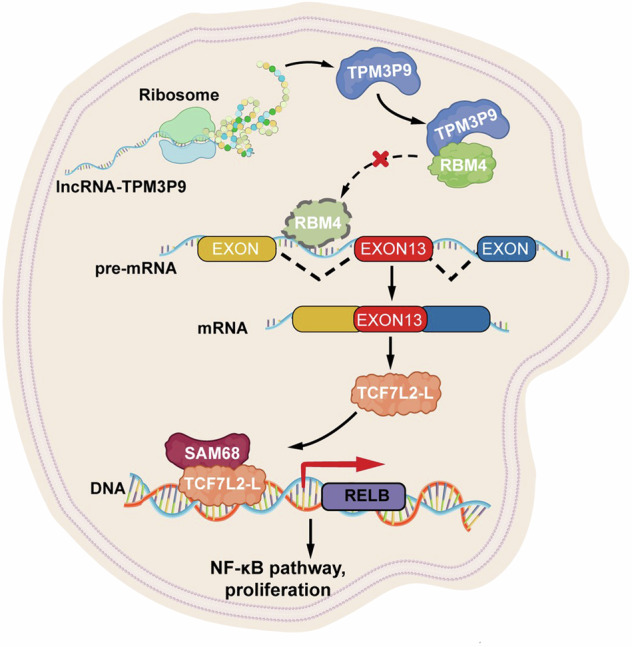
Hypothetical model. The microprotein TPM3P9 encoded by lncRNA-TPM3P9, is a tumor-promoting protein that drives kidney cancer proliferation. TPM3P9 interacts with the N-terminus of the RNA-binding protein RBM4, inhibiting RBM4’s regulatory effect on exon 13 skipping in TCF7L2 pre-mRNA, thereby facilitating the formation of the TCF7L2-L variant. TCF7L2-L further binds to the transcription factor SAM68 to promote the transcription of RELB, and thus activating the NF-κB signaling pathway and enhancing ccRCC cell proliferation. The diagram was edited using Adobe Illustrator

The human genome harbors a diverse and extensive array of lncRNA genes.^27^ Emerging evidence indicates that a relatively large number of lncRNAs are processed and spliced in a similar way to mRNAs to generate microproteins with oncogenic or anti-tumor activities.^28,29^ Several lncRNA-derived novel proteins have demonstrated promising prognostic and therapeutic value in the clinical management of malignant tumors.^10,30,31^ Previously, we performed profiling of full-length translating mRNA (RNC-seq) analysis to systematically identify >3000 lncRNAs that bind to the ribosome and thus could encode proteins. Here, we systemically integrated translatomic and proteomic data for cryptic lncRNAs and their encoded proteins obtained from CPTAC and SYSUCC samples and found that the lncRNA-encoded microprotein TPM3P9 was widely upregulated in multiple types of tumor tissues compared with the corresponding non-tumor tissues. Aberrantly high expression of TPM3P9 was associated with poor prognosis in renal cancer patients. TPM3P9 was found to interact with the splicing factor RBM4 to upregulate the oncogenic splice variant TCF7L2-L, which associates with SAM68 to transcriptionally induce RELB expression, thereby activating NF-κB signaling in ccRCC cells (Fig. 9).

LncRNAs regulate gene expression by interacting with chromatin-modifying proteins, transcription factors, RNA-binding proteins, and microRNAs.^27^ These interactions affect chromatin states, gene transcription,^27^ pre-mRNA splicing,^32^ mRNA stability,^33^ as well as protein translation and stability.^5,34^ In renal cell carcinoma, lncRNAs can act as both oncogenes and tumor suppressors. lncRNA FILNC1 serves as a negative regulator of RCC by inducing apoptosis through the downregulation of c-Myc protein translation.^35^ Additionally, lncRNA DRAIC regulates hnRNPA2B1 stability and promotes the instability of m6A-modified IGF1R, thereby inhibiting tumor progression.^36^ Recent research has increasingly shown that many lncRNAs can encode novel proteins that play critical roles in tumor development. These lncRNA-encoded proteins, with largely unknown functions, represent a biological treasure trove that may contain key molecules contributing to cancer progression. An initiative to characterize these emerging proteins has been proposed.

TPM3P9 represents the product of one of our ongoing efforts to identify new microproteins in ccRCC, which accounts for 70%-80% of renal cell carcinomas. Evidence has shown that lncRNA-derived proteins participate in DNA repair, RNA modification, and protein stability.^37–40^ However, the role of these uncharacterized proteins in RNA alternative splicing has been rarely explored. In colorectal cancer, LOC90024 was found to encode a small 130-aa protein, SRSP, which increases SRSF3-mediated RNA splicing of Sp4 to promote tumorigenesis.^41^ Here, we demonstrated that the novel protein TPM3P9 effectively modulated the alternative splicing of several genes. TCF7L2 functions as a central transcriptional regulator of the adipocyte metabolic program by directly regulating the expression of genes involved in lipid and glucose metabolism.^42,43^ TCF7L2 undergoes multiple alternative splicing events, among which exon skipping is the most well-studied in the context of oncogenesis.^44^ Alternative splicing of TCF7L2 modulates epithelial-mesenchymal transition *via* TGFβ-Smad3 signaling.^44^ TCF4N, an isoform of TCF7L2, binds to p65 and induces its phosphorylation at S536 to activate the NF-κB pathway in glioblastoma cells.^45^ In this study, we found that the TPM3P9-mediated long splice variant TCF7L2-L, which retains exon 13, interacted with SAM68 to upregulate RELB and activate NF-κB signaling. In addition, we further elucidated that only the TCF7L2 variant containing exon 13 promoted ccRCC cell proliferation and tumor growth.

Our data showed that the splicing factor RBM4 is essential for the TPM3P9-mediated inhibition of exon 13 skipping in TCF7L2. RBM proteins are a family of RNA-binding proteins that play important roles in the regulation of gene expression by modulating the synthesis or processing of RNAs, especially the alternative splicing of RNAs.^46^ RBM4 has been documented to suppress tumor progression in lung, breast, gastric, colorectal, and liver cancers.^25,47,48^ Similarly, we found that RBM4 was expressed at low levels in ccRCC and inhibited tumor growth. Interestingly, we found that TPM3P9 interacted with the N-terminus of RBM4, which is the core domain of RBM4, to bind to TCF7L2 pre-mRNA. In addition, we have performed further experiments to determine the interaction among TPM3P9, RBM4, and TCF7L2 pre-mRNA. The results showed that RBM4 interacts with TPM3P9 *via* its RRM1 domain and with TCF7L2 pre-mRNA *via* its RRM2 domain. Thus, we assume that the binding of TPM3P9 to RBM4 leads to a conformational change in the RBM4 protein, and to subsequently block its binding to TCF7L2 pre-mRNA. This observation suggests that TPM3P9 potentially impedes the interaction between RBM4 and TCF7L2 pre-mRNA, thereby facilitating the retention in exon 13 of TCF7L2 to promote renal cancer progression.

Although our findings show that RBM4 can bind to TCF7L2 pre-mRNA, the precise mechanisms by which RBM4 regulates the alternative splicing of TCF7L2 remain unclear. Additionally, the co-localization of RBM4 and TCF7L2 pre-mRNA within the cell, and whether TPM3P9 affects this co-localization, remain insufficiently explored. Determining whether TPM3P9, RBM4, and TCF7L2 pre-mRNA can form a complex will require further investigation. Moreover, antibody detection is the most direct method to verify the objective existence of novel proteins. However, this approach is often limited by long preparation times and inconsistent results, making it less suitable for high-throughput, large-scale identification of new proteins. Therefore, in future studies, methods like parallel reaction monitoring (PRM) mass spectrometry could be employed for rapid and accurate detection of microprotein expression in biological systems.

In conclusion, our findings demonstrate that the lncRNA-encoded protein TPM3P9 interacts with the RNA-binding protein RBM4 to inhibit exon skipping of TCF7L2, thereby increasing the expression of the TCF7L2-L variant, which promotes ccRCC cell proliferation through cooperation with SAM68 to transcriptionally upregulate RELB. Our study provides a new, comprehensive understanding of the molecular mechanisms underlying renal cancer development.

## Materials and methods

### Cell culture and treatments

ACHN, 786-O and HEK293 T cells were cultured in DMEM medium (Life Technologies); HK-2 cells were cultured in RPMI-1640 medium (Life Technologies); 1% penicillin-streptomycin (Life Technologies) and 10% FBS (Life Technologies) were added to all media; and cells were cultured at 37 °C with 5% CO_2_. All cells were authenticated by short tandem repeat profiling and tested negative for mycoplasma contamination. The plasmid constructs were obtained from IGE Biotechnology (Guangzhou, China), validated by DNA sequencing, and listed in Data S12. RNA interference and plasmid transfection were operated with Lipofectamine 3000 (Life Technologies) according to the manufacturer’s instructions.

### Clinical samples

Sixteen fresh paired ccRCC tissues and corresponding adjacent non-tumorous tissues, as well as postsurgical tissues from 385 patients with ccRCC, were collected at Sun Yat-sen University Cancer Center. Written informed consent was obtained from each participant. This study was approved by the institutional research ethics committee of Sun Yat-sen University Cancer Center (SL-B2022-288-01). The sample collection procedure was performed in accordance with the provisions of the 1975 Declaration of Helsinki.

### Immunofluorescence assays

After washing with PBS, cells were fixed with 4% paraformaldehyde and permeabilized with 0.5% Triton X-100. The samples were then blocked with 5% BSA for 1 h at room temperature, prior to incubation with primary antibodies overnight at 4 °C. After washing with 1 × TBST three times, the samples were incubated with fluorescent secondary antibodies (ABclonal Technology) at room temperature for 1 h, and nuclear staining was performed with DAPI (Keygen BioTECH, KGA1523-25) for 10 min. Then, the samples were visualized *via* confocal microscopy.

### Western blot

Western blot was performed as described previously.^49^ In brief, cells were lysed in cell lysis buffer, and a BCA Protein Assay Kit (Life Technologies) was used for protein quantification. Equal amounts of protein were separated by SDS-PAGE and transferred onto PVDF membranes (Bio-Rad). After being blocked with 5% nonfat milk at room temperature for 1 h, the membranes were incubated at 4 °C overnight with primary antibodies. The membranes were then washed with TBST three times prior to incubation with secondary antibodies for another 2 h. Immunoreactive bands were visualized by using the chemiluminescence western blot substrates (Bio-Rad) and imaged with a chemiluminescence imaging system (Tanon, China). The antibodies used in the experiments were as follows: anti-Flag (MBL, M185-3L), anti-Actin (Abclonal, AC050), anti-HA (CST, #3724), anti-RBM4 (Proteintech, 11614-1-AP), anti-SAM68 (Proteintech, 10222-1-AP), anti-TCF7L2-L (developed in-house), anti-TPM3P9 (developed in-house), anti-RELB (Abcam, ab33907), and anti-GFP (Proteintech, 50430-2-AP). HRP-conjugated goat anti-rabbit or anti-mouse IgG was used as the secondary antibody. The polyclonal antibodies against TPM3P9 were obtained from recombinant proteins inoculated rabbits. Anti-TPM3P9 antibodies were further purified using affinity chromatography on columns containing the corresponding recombinant proteins.

### T7E1 assay

The T7E1 assay was performed to assess the editing efficiencies of sgRNA.^50,51^ Briefly, the genomic DNA was extracted with the TIANamp Genomic DNA Kit (Tiangen, China), and the target sequence surrounding the cleavage site was amplified by PCR. PCR products were heated and annealed for the sake of forming heteroduplex DNA, then digested with T7 endonuclease 1 (Vazyme, China) at 37 °C for 15 min. DNA was separated by 2% agarose gel electrophoresis.

### Immunoprecipitation

For immunoprecipitation, cells were lysed with IP lysis buffer (50 mM Tris-HCl pH 7.4, 2 mM EDTA, 137 mM NaCl, 1% NP-40, and 10% glycerol) supplemented with protease inhibitor cocktail (Roche), and then incubated with primary antibodies overnight at 4 °C. The antibody-protein complexes in lysis buffer were incubated with 30 μL of Protein A/G Beads (Santa Cruz Biotechnology). After washing with IP Lysis Buffer five times, the protein-bound beads were mixed with 1 × loading buffer (Fdbio Science) and boiled for 10 min at 100 °C. Proteins in the samples were separated *via* SDS-PAGE.

### RNA pulldown

For the pulldown assay, TCF7L2 was transcribed in vitro using TranscriptAid T7 High Yield Transcription Kit (Thermo Scientific) according to the manufacturer’s instructions. Then, RNA was biotinylated with a Pierce RNA 30-End Desthiobiotinylation Kit (Thermo Scientific) under overnight incubation at 16 °C. After the biotinylated RNAs and streptavidin magnetic beads (Thermo Scientific) were mixed and incubated for 30 min at room temperature, the cell lysates and RNase inhibitor were added, and the mixture was incubated for another hour at 4 °C with rotation. The magnetic bead complexes were subsequently washed three times with wash buffer. After the complexes were boiled in SDS buffer, proteins were separated *via* SDS-PAGE or further digested with trypsin and identified *via* mass spectrometry with Orbitrap Fusion Lumos mass spectrometer.

### RNA immunoprecipitation (RIP)

RNA immunoprecipitation (RIP) assays were performed with an RNA immunoprecipitation kit following the manufacturer’s protocol (Bersinbio, China). Briefly, ACHN and 786-O cells were lysed in RIP lysis buffer supplemented with protease and RNase inhibitors. Protein A/G magnetic beads conjugated to rabbit immunoglobulin G or an anti-RBM4 antibody were added and incubated with the cell lysates at 4 °C overnight. After six times washes, the RNA-protein complexes were incubated with proteinase K. Finally, RNA was extracted using the phenol-chloroform method and analysed *via* qRT-PCR.

### Immunohistochemistry

Tissue microarrays (TMAs) containing RCC tissues and adjacent normal renal tissues were used to evaluate the expression levels of TPM3P9, RELB, SAM68, and TCF7L2 *via* immunohistochemistry (IHC). The sections were deparaffinized with xylene and rehydrated. Antigen retrieval was performed with antigen retrieval buffer by boiling for 2.5 min in a pressure cooker. Then, the sections were treated with 3% hydrogen peroxide to quench endogenous peroxidase activity. After the sections were incubated with blocking buffer to block nonspecific binding, they were incubated with primary antibodies overnight at 4 °C. After three washes with PBS, the sections were incubated with an HRP-conjugated anti-rabbit or mouse secondary antibody (PV-6000, Zhongshan Golden Bridge Biotechnology, Beijing) for 20 min, prior to washing with PBS. DAB chromogen substrate was added to the stained sections, and the staining intensity was evaluated under a microscope. Two investigators independently reviewed and scored the degree of immunostaining in the sections, based on the percentage of positively stained cells and the staining intensity.

### Dual-luciferase reporter assay

For the RELB promoter luciferase reporter assay, the core promoter of the RELB gene was synthesized, cloned and inserted into the pGL3-basic firefly luciferase reporter vector. The pRL-TK vector was used as a control. Then cells were transfected with the RELB reporter vector and pRL-TK as the internal control. Forty-eight hours after transfection, luciferase activity was measured with a dual luciferase assay system (Promega, USA). Firefly luciferase activity was normalized to Renilla luciferase activity.

### EdU assay

Cells were cultured in a 12-well plate and treated with 100 μL of medium containing 20 μM EdU. After incubation at 37 °C in 5% CO_2_ for 2 h, the cells were fixed with 4% paraformaldehyde for 30 min and incubated with 0.5% Triton-X-100 in PBS for 20 min. Nuclei were stained with DAPI. Images of five randomly selected fields of view in each group were acquired with a fluorescence microscope and analysed with ImageJ. The proliferation rate was calculated according to the manufacturer’s instructions.

### Animal experiments

The four-week-old male BALB/c nude mice used in this study were purchased from GemPharmatech (Guangzhou, China). The animal studies were approved by the Ethics Committee for Animal Experiments of Jinan University (IACUC-20220411-11), and all of the animal research was conducted in compliance with the Guidance for the Care and Use of Laboratory Animals of Jinan University. All animals were maintained under specific-pathogen-free (SPF) conditions, with the controlled environmental temperature of 23 ± 1 °C, a 12-h light/dark cycle, and relative humidity levels maintained at 55% ± 5%. Animals were randomly assigned to the control group and the experimental group. For the xenograft experiment, each stably transfected ACHN cells (5 million cells resuspended in a 100 μL volume) were subcutaneously injected into five mice. Xenografts were measured every 5 days with digital calipers, and tumor volumes were calculated using the following equation: volume = 1/2 (length × width^2^). Thirty-five days later, the mice were sacrificed, and the tumor volumes were calculated. Samples were embedded in paraffin for hematoxylin and eosin (HE) staining and immunohistochemical staining.

### LEP identification

Raw pan-cancer data from the following CPTAC study IDs were downloaded: PDC000120 (breast), PDC000411 (kidney), PDC000116 (colon), PDC000221 (head and neck), PDC000198 (liver), PDC000234 (lung), PDC000110 (ovary), PDC000341 (pancreas), and PDC000125 (uterus). After the raw files were downloaded, the reference library from a previous study was used for the data search. For TMT data, including breast cancer, colon cancer, head and neck cancer, liver cancer, lung cancer, ovarian cancer, uterine cancer data, Proteome Discoverer (Thermo Scientific) was used for the data search. For DIA data, including kidney cancer and pancreatic cancer data, Spectronaut (Biognosys) was used for the data search. In the data obtained, proteins with no missing values in >30% of the tumor samples or >30% of the non-tumor samples were considered to be LEPs.

### Mass spectrometry analysis

LC-MS/MS analysis for protein identification was performed on an Orbitrap Fusion Lumos mass spectrometer (Thermo Scientific). In brief, samples were lysed in lysis buffer supplemented with protease inhibitors and subjected to tryptic digestion *via* the FASP method. The tryptic peptides were solubilized with 0.1% formic acid and identified *via* mass spectrometry. Proteome Discoverer (Thermo Scientific) and Spectronaut (Biognosys) software were used for quantification.

### Ribosome—nascent chain complex extraction

HK-2, ACHN and 786-O cells were pretreated with 100 μg/mL cycloheximide for 15 min, and then washed with precooled PBS twice. Then, 2 mL of lysis buffer (20 mM HEPES-KOH, 200 mM KCl, 15 mM MgCl_2_, 100 μg/mL cycloheximide, 2 mM dithiothreitol, and 1% Triton X-100) was added to the cells, which were lysed on ice for 30 min. The cells were collected and centrifuged at 12,000 × g for 10 min at 4 °C. The supernatant was transferred to the surface of a 20 mL volume of 30% sucrose buffer, and RNC was pelleted by ultra-centrifugation at 185,000 × g for 5 h at 4 °C. After the complexes were washed with lysis buffer, RNA was extracted using the phenol-chloroform method and subjected to RNA-Seq analysis.

### Statistical analysis

For differential expression analysis in the CPTAC cohort, the proteomic data with no missing values in >50% of the tumor samples and >50% of the non-tumor samples were used as input data. Computational and statistical analyses were performed using GraphPad Prism 8.0 and R software (version 4.1.2). LEPs with *p* < 0.05 and |fold change | ≥ 1.5 were considered to be significant LEPs. Data were analyzed by paired/unpaired two-tailed Student’s *t*-tests and two-way ANOVA. The Mann–Whitney U test was performed for data that did not follow a normal distribution. Survival data were analyzed by the log-rank test. For all analyses, a *p* < 0.05 was considered to indicate statistical significance. The network was created using Cytoscape software and the STRING database.

## Supplementary information

Supplementary

western blot raw data

Supplementary Data S1

Supplementary Data S2

Supplementary Data S3

Supplementary Data S4

Supplementary Data S5

Supplementary Data S6

Supplementary Data S7

Supplementary Data S8

Supplementary Data S9

Supplementary Data S10

Supplementary Data S11

Supplementary Data S12

## Data Availability

The RNA-seq data for cells with overexpression of TPM3P9 and overexpression of different TCF7L2 variants have been deposited in the Sequence Read Archive database (SRA database, https://www.ncbi.nlm.nih.gov/sra/) under accession number PRJNA1057324 and PRJNA1057325, respectively. The translatome data of HK-2 cell line as well as the ccRCC cell lines ACHN and 786-O are available in the SRA database under the accession number PRJNA1066097. The mass spectrometry data have been deposited to the ProteomeXchange Consortium (https://pdc.cancer.gov/pdc/) *via* the PRIDE database, including the RNA-pulldown MS of TCF7L2 pre-mRNA (PXD048312), the Co-IP MS of TCF7L2-S and TCF7L2-L variants (PXD048102), the Co-IP MS of TPM3P9 (PXD048101). The proteomics data of 58 pan-cancer samples were deposited to the ProteomeXchange Consortium (PXD048734). All other relevant data are available from the authors.
