# Supplementary material for: The cryptic lncRNA-encoded microprotein TPM3P9 drives oncogenic RNA splicing and tumorigenesis
Source: Signal Transduct Target Ther. 2025 Jan 27;10:43. doi: 10.1038/s41392-025-02128-8 (PMC11770092; doi:10.1038/s41392-025-02128-8)
Supplement: Supplementary file 1 — Supplementary [file 41392_2025_2128_MOESM1_ESM.docx]

Supplementary Materials for

**The cryptic lncRNA-encoded microprotein TPM3P9 drives oncogenic**

**RNA splicing and tumorigenesis**

Kun Meng, Yuying Li, Xiaoyi Yuan, Hui-Min Shen, Li-Ling Hu, Danya Liu, Fujin Shi, Dandan Zheng, Xinyu Shi, Nengqiao Wen, Yun Cao, Yun-Long Pan, Qing-Yu He, Chris Zhiyi Zhang

Correspondence: zhangzy@jnu.edu.cn (CZ Zhang); tqyhe@email.jnu.edu.cn (QY He)

**This PDF file includes:**

Figures. S1 to S10

Tables S1 to S5

Captions for Data Sl to S12

**Other Supplementary Materials for this manuscript include the following:**

Data S1 to S12 (separate file)

**Supplementary Figure 1**


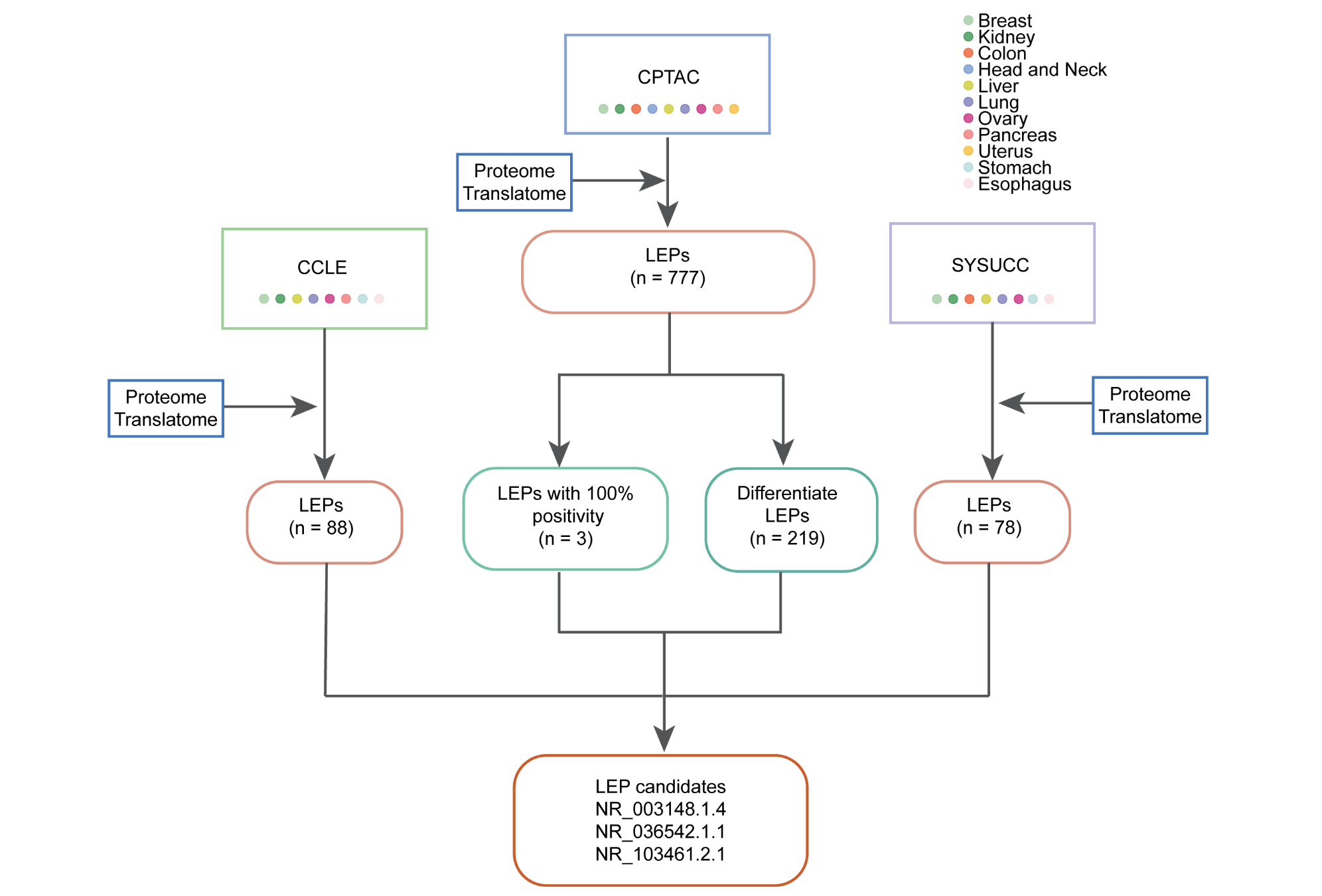


**Figure S1.** The workflow to screen candidate microproteins.

**Supplementary Figure 2**


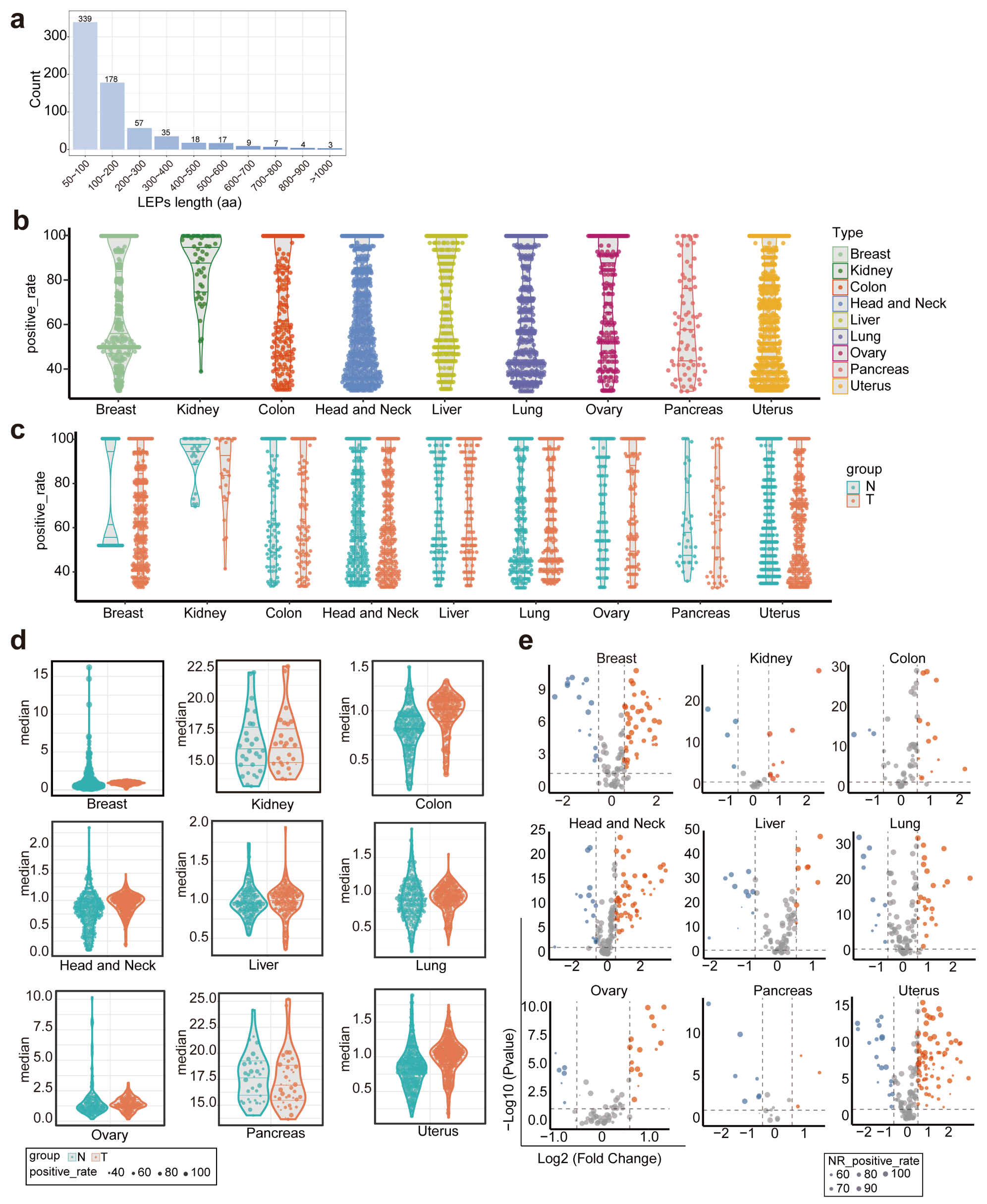


**Figure S2. Identification and validation of microproteins encoded by lncRNA in human cancers. (a)** The length distribution of the lncRNA-encoded proteins in tumor tissues. **(b)** Distribution of the positive rate of LEPs identified in the CPTAC cohort. **(c)** The positive rate of LEPs was identified across 9 cancer types between cancer and noncancerous samples. **(d)** The median expression of LEPs between cancer (orange) and noncancerous (blue) samples. The size of the dot indicates the positive rate of LEPs. **(e)** Volcano plot of the difference between cancer and noncancerous samples of LEPs identified. The size of the dot indicates the positive rate of LEPs, with red representing up-regulation in cancer samples and blue representing down-regulation.

**Supplementary Figure 3**


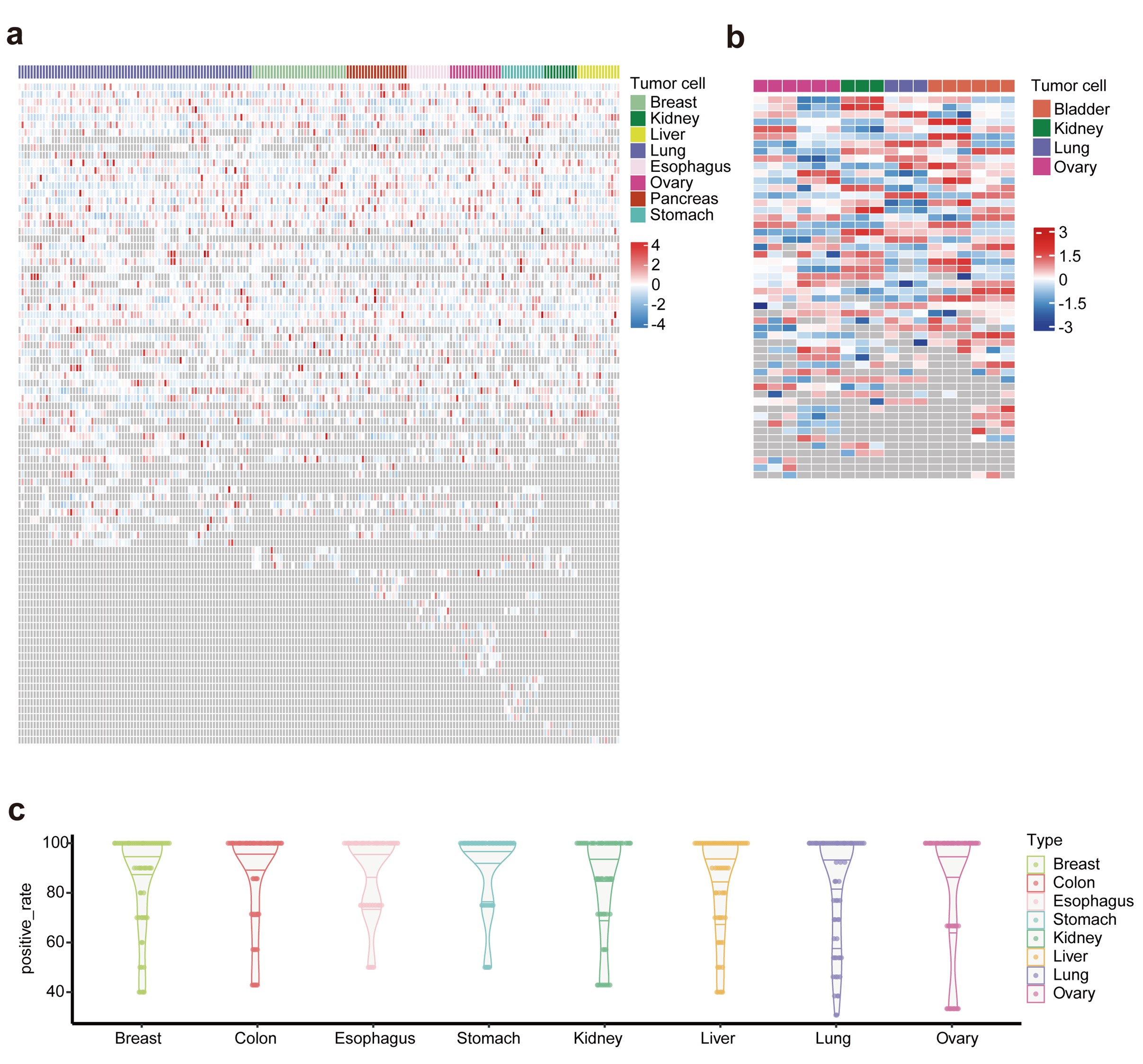


**Figure S3. Microproteins exhibit widespread expression across a variety of human tumor cells. (a)** Proteomics analysis of lncRNA-encoded proteins (LEPs) was conducted using data from the Cancer Cell Line Encyclopedia (CCLE). **(b)** Proteomics analysis of lncRNA-encoded proteins with tumor cells preserved in our laboratory. **(c)** Distribution of the positive rate of LEPs identified in the SYSUCC cohort.

**Supplementary Figure 4**


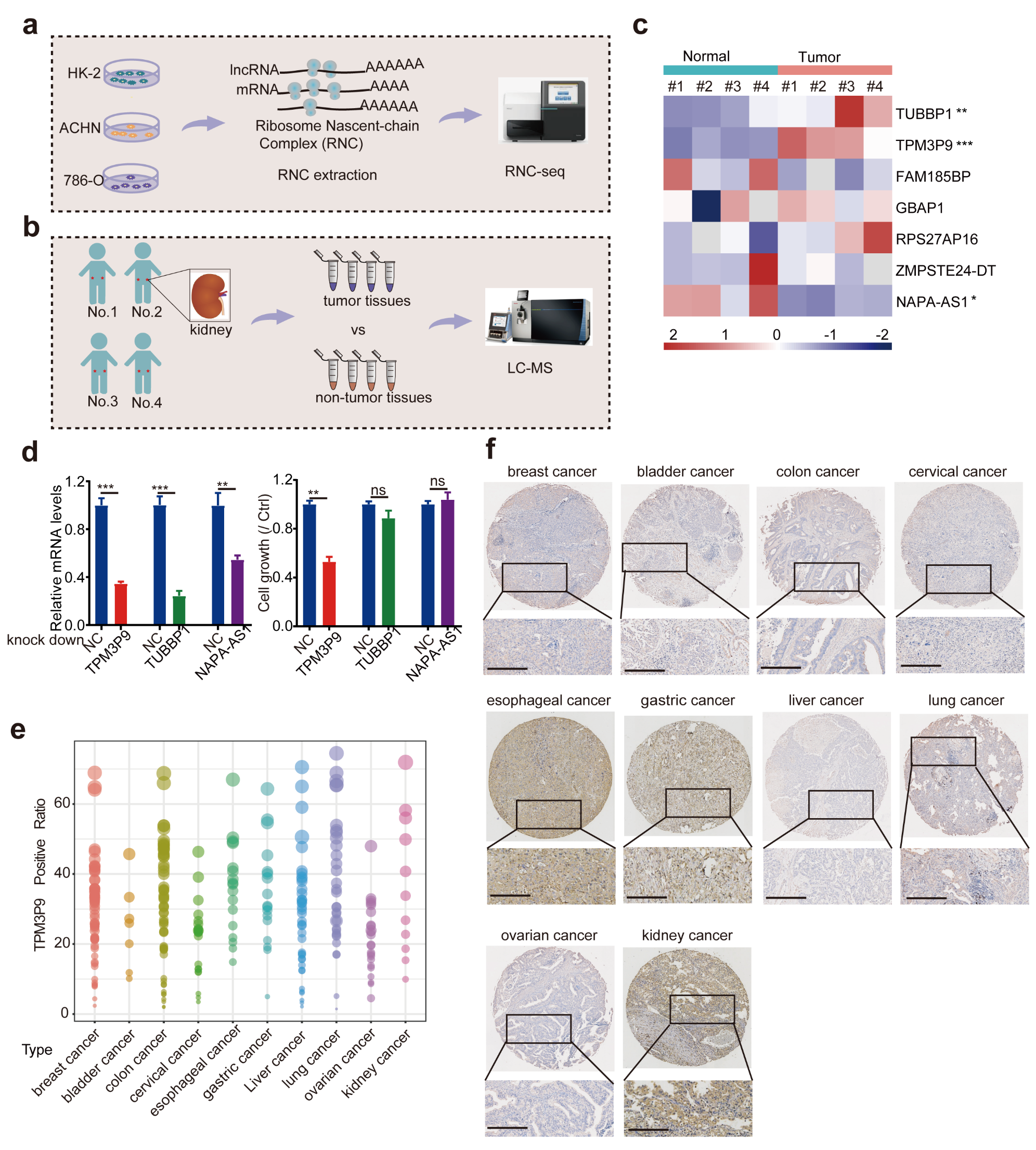


**Figure S4. Novel microprotein TPM3P9 is upregulated in human cancers. (a)** The workflow of RNC-seq carried on ccRCC cells ACHN and 786-O, along with renal tubular epithelial cell HK-2. **(b)** The proteomic profiling workflow of four matched ccRCC tissues and the corresponding adjacent normal tissues by using high-resolution mass spectrometry. **(c)** LEPs encoded by lncRNA-TUBBP1, lncRNA-TPM3P9, and lncRNA-NAPA-AS1 showed significantly differential expression in ccRCC compared to normal tissues. **(d)** Cell growth assays showing TPM3P9 but not TUBBP1 and NAPA-AS1 knockdown inhibited the proliferation of ACHN cells. **(e-f)** Immunohistochemistry showing TPM3P9 had universal expression in nearly all types of cancers in SYSUCC cohorts. Scale bar, 200 μm.

**Supplementary Figure 5**


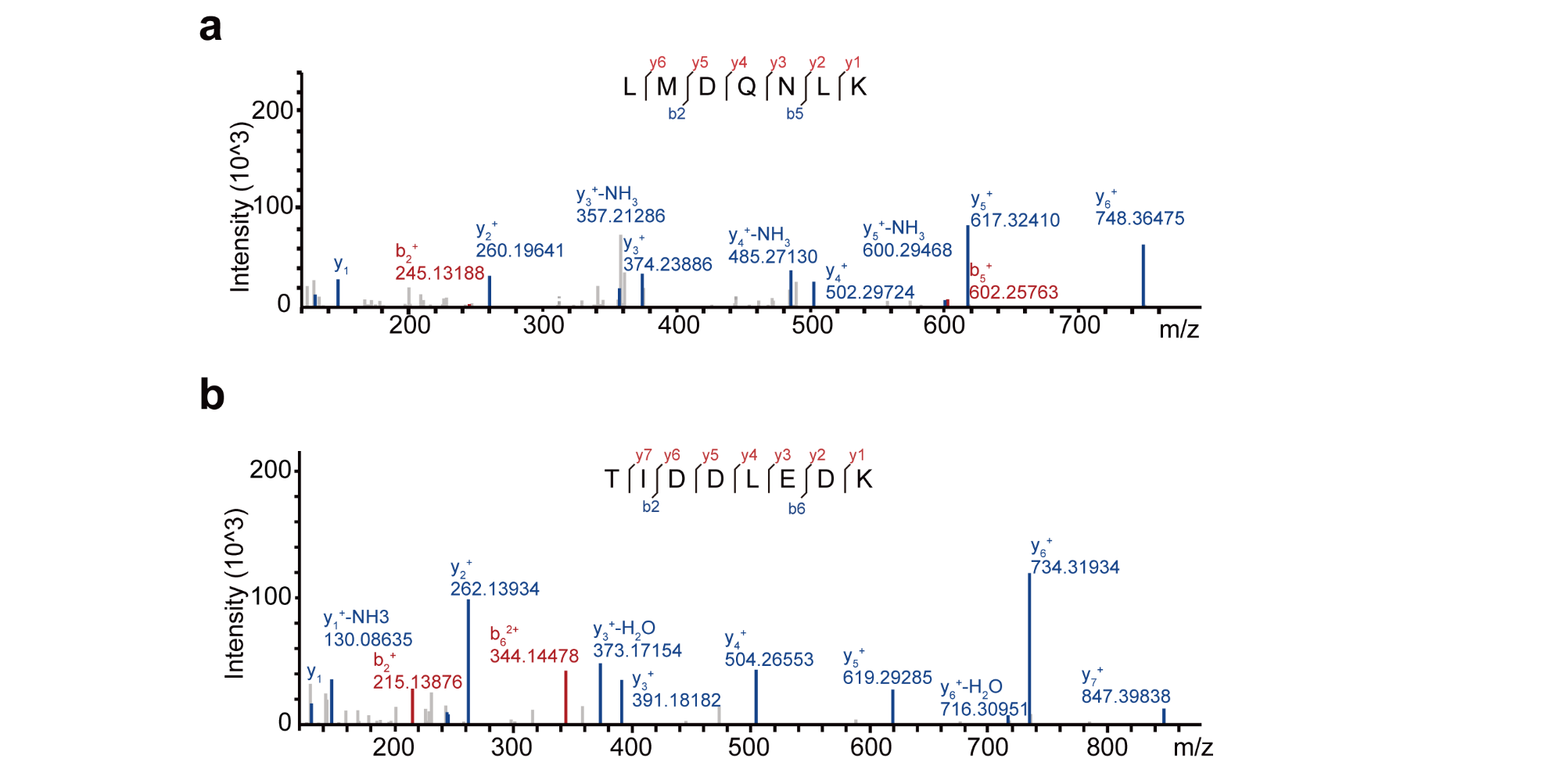


**Figure S5. The novel microprotein TPM3P9 is upregulated in ccRCC.** **(a-b)** Two unique peptides of TPM3P9 were identified by shotgun MS.

**Supplementary Figure 6**

**
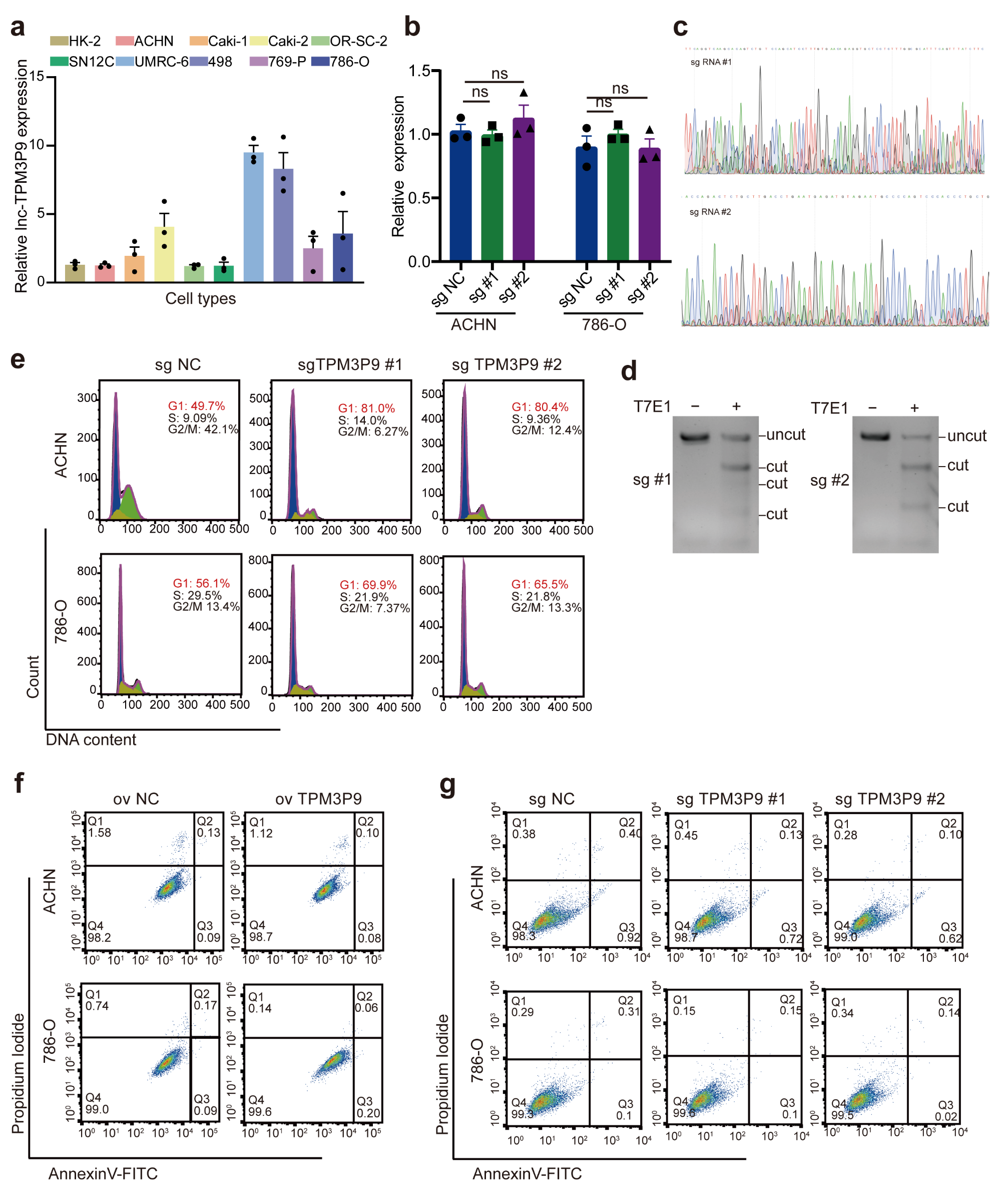
**

**Figure S6. TPM3P9 induced G1 phase arrest but had a limited effect on cell apoptosis. (a)** The qRT-PCR was conducted to measure the expression levels of lncRNA-TPM3P9 in several ccRCC cell lines. **(b)** The qRT-PCR analysis demonstrates that sgRNA does not affect the RNA expression levels of TPM3P9 in ccRCC cells. **(c)** The sequencing results showed mixed peaks with nucleotide mutations or deletions downstream of the sgRNA sequences. (**d**) The validation of DNA editing by sgRNA#1 and sgRNA#2 using a T7 endonuclease I assay. **(e)** Cell cycle assays demonstrated TPM3P9 silence markedly induced G1 phase arrest in ccRCC cells. **(f-g)** Cell apoptosis experiments showing overexpression (f) or knockdown (g) of TPM3P9 had limited effect on ccRCC cell apoptosis.

**Supplementary Figure 7**


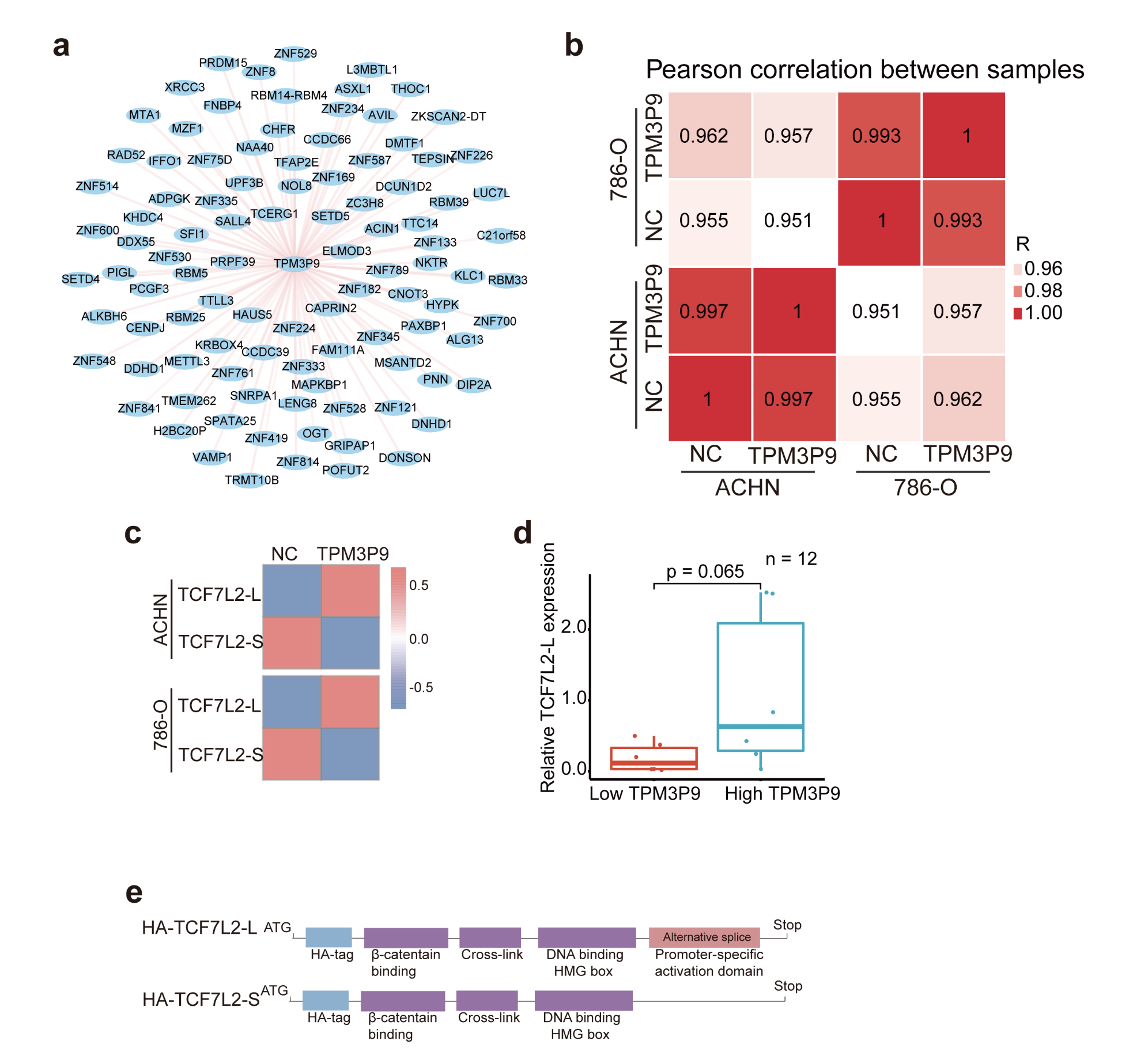


**Figure S7. TPM3P9 regulates the alternative splicing of TCF7L2 pre-mRNA. (a)** The co-expression network analysis showing the proteins co-expressing with TPM3P9. **(b)** Pearson correlation between RNA-seq results of ccRCC cells with control or TPM3P9 overexpression. **(c)** RNA-seq results indicated that ectopic expression of TPM3P9 increased the expression of long splicing variant TCF7L2-L. **(d)** Expression analysis of TPM3P9 protein and TCF7L2-L mRNA in 12 ccRCC tissue samples. **(e)** Schematic representation of the vectors encoding TCF7L2-L variant and TCF7L2-S variant, respectively.

**Supplementary Figure 8**

**
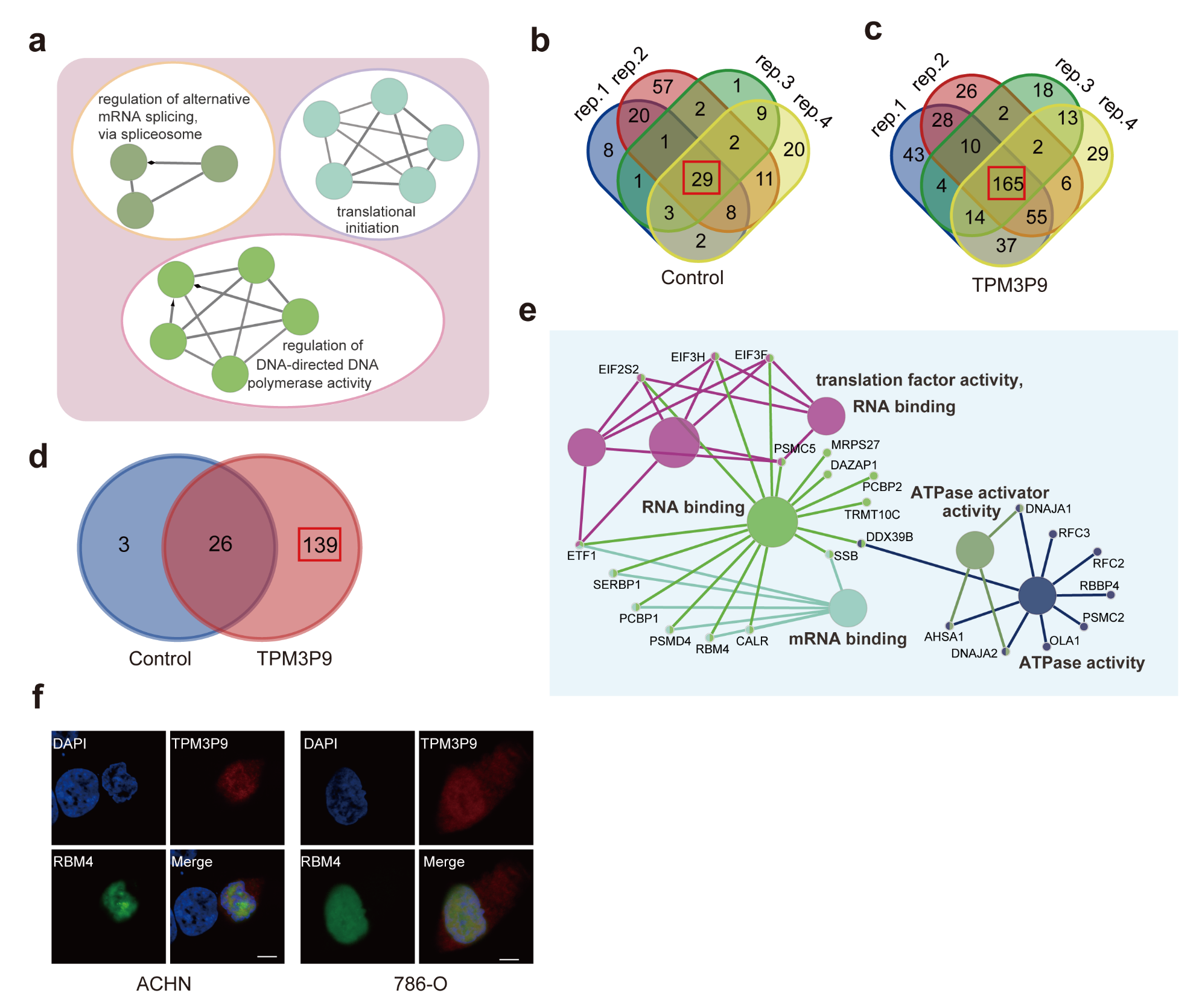
**

**Figure S8. TPM3P9 had an interaction with RNA-binding protein RBM4. (a)** Gene Ontology analysis revealed that the RNA splicing pathway was enriched in the proteins interacting with TCF7L2 pre-mRNA. **(b)** Venn diagram showing 29 proteins were identified in four independent biological replicates of control, using Flag-specific antibody. **(c)** Venn diagram showing 165 proteins were identified in four independent biological replicates of TPM3P9 overexpression, using Flag-specific antibody. **(d)** Venn diagram showing that 139 proteins specifically had an interaction with TPM3P9, compared with the control. **(e)** Gene Ontology analysis revealed that the RNA splicing pathway was enriched in the proteins interacting with TPM3P9. **(f)** Immunofluorescence assays validated the interaction between RBM4 and TPM3P9 in the nucleus of ccRCC cells. Nuclei were stained with DAPI (blue), TPM3P9 (red), and RBM4 (green). Scale bar, 5 μm.

**Supplementary Figure 9**

**
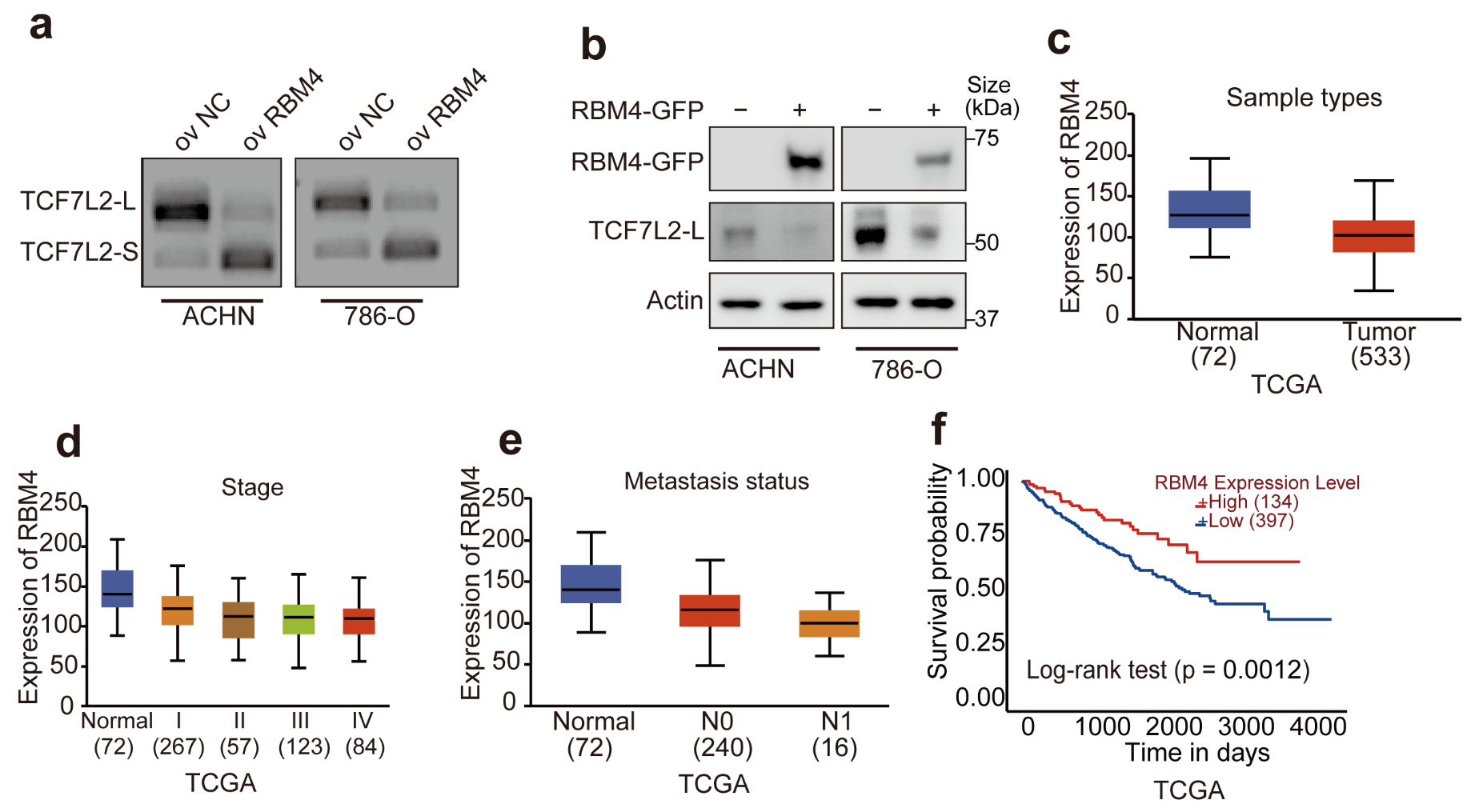
**

**Figure S9. High RBM4 expression inhibits the formation of the TCF7L2-L variants and correlates with favorable clinical characteristics of ccRCC patients. (a)** Overexpression of RBM4 increased the RNA expression of the TCF7L2-S variant but decreased the RNA expression of the TCF7L2-L variant in ccRCC cells. **(b)** Western blot showing RBM4 overexpression decreased the protein expression of the TCF7L2-L variant in ccRCC cells. **(c)** The TCGA database revealed that RBM4 was significantly less expressed in ccRCC tissues, compared to the nontumor tissues. **(d-e)** The TCGA database displayed that low RBM4 expression was significantly correlated with high clinical stage (d) and more lymph node metastasis (e). **(f)** Kaplan-Meier survival analysis shows ccRCC patients with high RBM4 expression were accompanied by favorable prognosis in the TCGA cohort (log-rank test). UALCAN (https://ualcan.path.uab.edu/) was used for expression validation and survival analysis.

**Supplementary Figure 10**

**
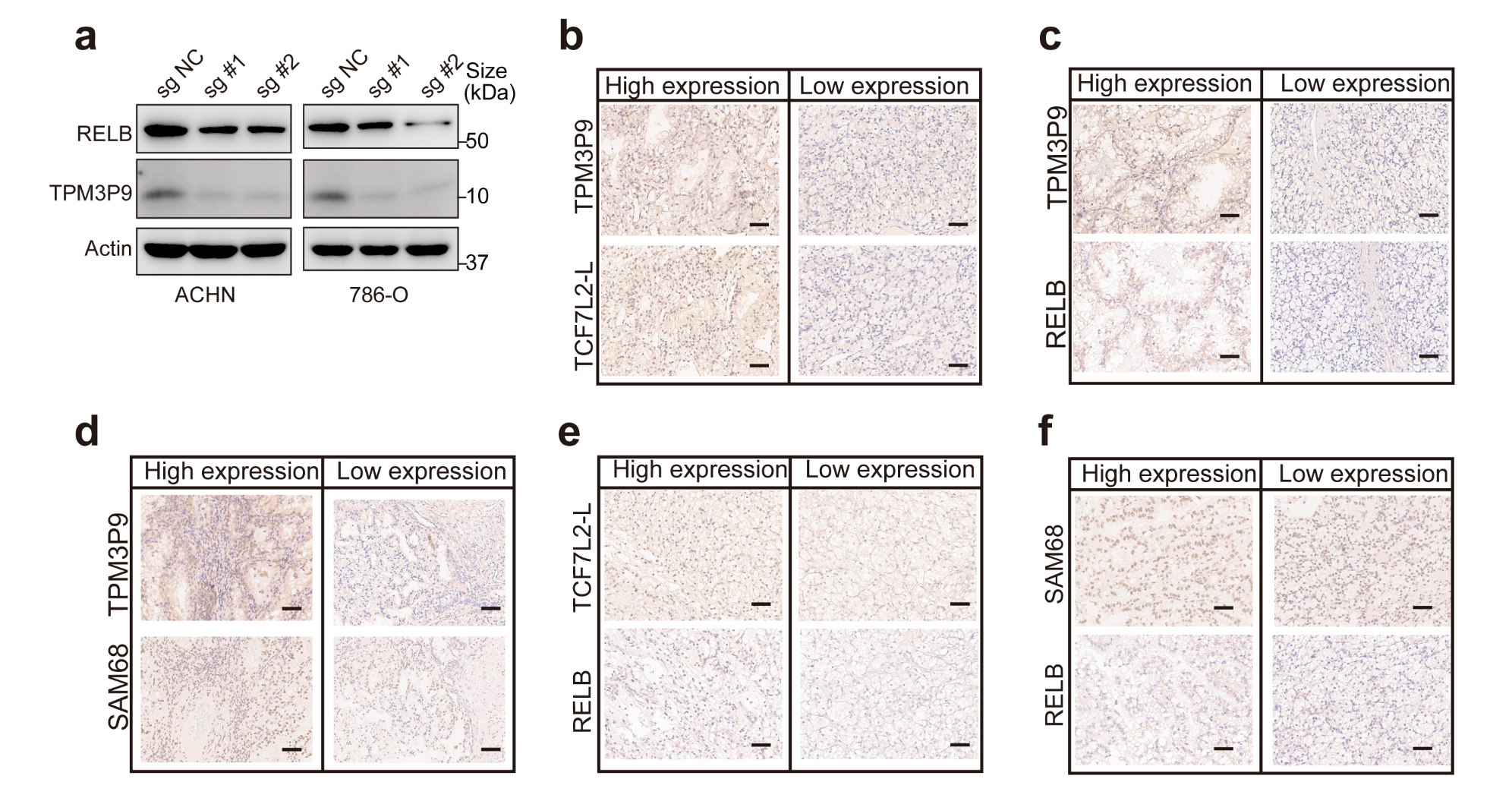
**

**Figure S10. The correlation analysis of TPM3P9, TCF7L2-L, RELB and SAM68, and RELB expression in the SYSUCC cohort. (a)** Western blot analysis confirmed that knockdown of the TPM3P9 inhibited RELB expression in ccRCC cells. **(b-f)** Immunohistochemistry assays performed on the SYSUCC cohort with 385 clinical samples showing the expression correlation of the TCF7L2-L with TPM3P9 (b), RELB with TPM3P9 (c), SAM68 with TPM3P9 (d), RELB with TCF7L2-L (e), RELB with SAM68(f). Scale bar, 50 μm.

| **Supplementary Table 1. Clinical characteristics of patients with ccRCC in this study** | | |
| --- | --- | --- |
| **Characteristics** | **Total** | **No. (%)** |
| Patients | 385 |  |
| Age at diagnosis (years) | 52 (25-82) |  |
| Gender |  |  |
| Male | 267 | 69.4 |
| Female | 118 | 30.6 |
| Fuhrman grading |  |  |
| 1 | 14 | 3.6 |
| 2 | 205 | 53.2 |
| 3 | 155 | 40.3 |
| 4 | 11 | 2.9 |
| Necrosis |  |  |
| No | 317 | 82.3 |
| Yes | 68 | 17.7 |
| T stage |  |  |
| 1 | 281 | 73 |
| 2 | 50 | 13 |
| 3 | 51 | 13.2 |
| 4 | 3 | 0.8 |
| Lymph nodes invasion |  |  |
| No | 371 | 96.4 |
| Yes | 14 | 3.6 |
| Distal metastasis |  |  |
| No | 370 | 96.1 |
| Yes | 15 | 3.9 |

**Supplementary Table 2. Pre-mRNA TCF7L2 interacted proteins identified by RNA-pulldown combined with MS**

| **Protein** | **Protein** | **Protein** |
| --- | --- | --- |
| RFC3 | EIF3M | SSB |
| HNRNPDL | LSM14B | KHDRBS1 |
| HNRNPA3 | HNRNPD | DAZAP1 |
| ILF2 | RTCA | CMAS |
| PPP1CC | ACOT9 | LYAR |
| ALDOA | TFAP2D | EIF4A1 |
| KRT19 | SLC25A3 | PCBP1 |
| RFC5 | HNRNPA2B1 | PA2G4 |
| QKI | EIF3F | PACSIN3 |
| KRT15 | HNRNPH2 | POLR1C |
| DRG1 | PHF6 | TARDBP |
| CSNK1A1L | TP53 | ZC3H15 |
| FEN1 | LHX6 | RBMX |
| DEK | SERBP1 | YBX3 |
| RBM4 | SRSF6 | DDX39A |
| RAE1 | HNRNPF | EIF2S2 |
| EIF3H | PCBP2 | RFC4 |
| STRAP | MAZ | ING3 |
| HNRNPA1 | ACAT1 | RFC2 |
| NANS | DNAJA1 | POGLUT1 |
| BUB3 | EIF2B2 | RPL3 |
| TIAL1 | KCTD16 |  |

**Supplementary Table 3. TPM3P9 binding proteins identified by Co-IP MS**

| **Protein** | **Protein** | **Protein** |
| --- | --- | --- |
| RBM4 | SSB | ZNF24 |
| PSAT1 | DAZAP1 | CKMT1A |
| GOT2 | ERLIN2 | LYAR |
| CALU | RCN1 | SERPINH1 |
| ACTR3 | ALKBH5 | GOT1 |
| PRMT1 | DCTN2 | PDHB |
| NELFE | ACTR1B | RFC2 |
| DEK | HM13 | LAMP2 |
| MRPS9 | PSMC5 | RBBP7 |
| ANXA2 | CTSD | MAPK3 |
| TRMT10C | SEPTIN2 | TRIP13 |
| MRPL37 | PHF6 | WDR12 |
| RBM17 | IDH1 | PSMD4 |
| CNP | EIF2S2 | NSFL1C |
| LANCL1 | ERP44 | KRT17 |
| PAFAH1B1 | PSMC6 | FAM50A |
| ACTR2 | SAE1 | MRPS22 |
| DDX39B | PTGES2 | ACTR1A |
| ALDOA | RCC1 | ADRM1 |
| HDHD5 | TBL2 | IDH3B |
| EIF3H | COPS3 | MRPS27 |
| RFC3 | SERPINB12 | CCDC51 |
| TALDO1 | UQCRC2 | PSMC4 |
| PSMC3 | PSMD11 | ACADM |
| CSNK2A2 | NUDC | IGHA1 |
| PDHA1 | SERPINB3 | APMAP |
| STRAP | OLA1 | GNB2 |
| COPS4 | PCBP1 | GLRX3 |
| BUB3 | VAT1 | CALR |
| EIF3F | FEN1 | PSMD13 |
| TMEM43 | PDIA6 | DRG1 |
| TXNDC5 | CD2BP2 | DNAJA2 |
| PSMD6 | GNAI3 | ARFIP2 |
| RNH1 | PPA2 | ACAT1 |
| ACAT2 | WDR18 | TIMM50 |
| HACD3 | AGK | ERLIN1 |
| ALDOC | STOML2 | ST13 |
| PSMD7 | ETF1 | CDC37 |
| PRKAR2A | BSG | OAT |
| PSMC2 | PGK1 | PCBP2 |
| NAP1L1 | HIBCH | EPM2A |
| ARMCX3 | AHSA1 | QKI |
| CSNK2A1 | MRPS31 | MAT2A |
| BZW1 | PLIN3 | DNAJA1 |
| FDPS | ACADSB | HAT1 |
| RBBP4 | PA2G4 |  |
| PPID | SERBP1 |  |

**Supplementary Table 4. Pre-mRNA TCF7L2 interacted proteins predicted by catRAPID**

| **Protein** | **Protein** | **Protein** |
| --- | --- | --- |
| SAFB2 | U2AF1 | YTHDF2 |
| DHX9 | TBRG4 | RBM23 |
| FAM120A | SRSF5 | CELF1 |
| HNRNPU | RBM4 | U2AF2 |
| KHSRP | IGF2BP2 | HNRNPK |
| DDX54 | MSI2 | SRSF10 |
| ACIN1 | TRA2A | DDX55 |
| AGGF1 | FMR1 | SRSF9 |
| RBM15B | HNRNPUL1 | PTBP1 |
| DDX24 | NCBP2 | RBM22 |
| AKAP1 | DDX19B | SRP68 |
| RBM6 | ALYREF | ZNF622 |
| DDX58 | SRSF3 | RBM24 |
| RBM25 | FUS | PTBP3 |
| SUPV3L1 | METTL14 | SRSF1 |
| SUGP2 | LIN28A | METTL3 |
| ADAR | HNRNPH3 | FUBP3 |
| XPO5 | SRSF8 | SND1 |
| IGHMBP2 | ZNF346 | IFIH1 |
| ESRP1 | RBM47 | DGCR8 |
| AGO1 | SF3A3 | FASTKD2 |
| AGO2 | SRSF4 | HLTF |
| YBX1 | RBM14 | RBM28 |
| YTHDC2 | FXR1 | AUH |
| RC3H1 | DDX6 | LARP4B |
| KHDRBS2 | ELAVL2 | NSUN2 |
| TNRC6A | NOVA2 | PPRC1 |
| PUM2 | GTF2F1 | SAMD4A |
| BCCIP | CPSF6 | QKI |
| YTHDC1 | ELAVL3 | RBM4B |
| RBM15 | TIAL1 | NOL12 |
| FXR2 | DDX59 | FUBP1 |
| KHDRBS3 | CPEB2 | TRNAU1AP |
| PABPC1 | TARBP2 | HNRNPC |
| PUM1 | HNRNPH2 | HNRNPM |
| SFPQ | WTAP | RBMY1A1 |
| BUD13 | YTHDF1 | HNRNPCL1 |
| CAPRIN1 | NOVA1 | SSB |
| G3BP1 | PCBP2 | BOLL |
| SRP54 | HNRNPH1 | MSI1 |
| ILF3 | SRSF7 | SNRPA |
| ZFP36 | LIN28B | CDC40 |
| SRP19 | IGF2BP3 | PPIL4 |
| PCBP1 | RBM45 | ZFP36L2 |
| LARP4 | CSTF2T | RBM41 |
| SUB1 | TAF15 | GRWD1 |
| SRSF6 | ELAVL4 | HNRNPLL |
| ELAVL1 | HNRNPF | HNRNPL |
| TIA1 | HNRNPA3 | GPKOW |
| MBNL2 | RO60 | NUP42 |
| ZNF326 | HNRNPA1 | EWSR1 |
| HNRNPA2B1 | DAZ3 | RBMS1 |
| RALY | YBX3 | ILF2 |
| MBNL1 | SRSF2 | CNOT4 |
| DHX58 | YTHDF3 |  |

**Supplementary Table 5. TCF7L2-L interacted proteins identified by Co-IP MS**

| **Gene name** | **Description** |
| --- | --- |
| SAM68 | KH domain-containing, RNA-binding, signal transduction-associated protein 1 |
| MYBBP1A | Myb-binding protein 1A |
| HNRNPA3 | Heterogeneous nuclear ribonucleoprotein A3 |
| RBM26 | RNA-binding protein 26 |
| HMGB2 | High mobility group protein B2 |
| MYH14 | Myosin-14 |
| TPM2 | Tropomyosin beta chain |
| UBA52 | Ubiquitin-60S ribosomal protein L40 |
| PRKDC | DNA-dependent protein kinase catalytic subunit |
| NCCRP1 | F-box only protein 50 |
| TGM1 | Protein-glutamine gamma-glutamyltransferase K |
| MYO1C | Unconventional myosin-Ic |
| CAPZB | F-actin-capping protein subunit beta |
| ALDOC | Fructose-bisphosphate aldolase C |
| ACAP1 | Arf-GAP with coiled-coil, ANK repeat and PH domain-containing protein 1 |
| KRT13 | Keratin, type I cytoskeletal 13 |
| MDH2 | Malate dehydrogenase, mitochondrial |
| YWHAZ | 14-3-3 protein zeta/delta |
| KRT4 | Keratin, type II cytoskeletal 4 |
| KARS | Lysine--tRNA ligase |
| VDAC2 | Voltage-dependent anion-selective channel protein 2 |
| TGM3 | Protein-glutamine gamma-glutamyltransferase E |
| AIMP2 | Aminoacyl tRNA synthase complex-interacting multifunctional protein 2 |
| KRT80 | Keratin, type II cytoskeletal 80 |
| ANXA1 | Annexin A1 |
| OAT | Ornithine aminotransferase, mitochondrial |
| AHSG | Alpha-2-HS-glycoprotein |

Data S1 to S12 (separate file)

Data S1. LEPs were identified in tumor and non-tumor samples for each cancer type.

Data S2. Distribution of LEPs identified in the CPTAC cohort on chromosomes.

Data S3. The positive rate and median expression of LEPs were identified for 9 cancer types.

Data S4. Unique LEPs were identified in each cancer type.

Data S5.The positive rate and median expression values of the three LEPs were identified in all 9 cancer types.

Data S6. Difference analysis results in 9 cancer types.

Data S7. LEPs were differentially expressed between tumor and non-tumor samples.

Data S8. LEPs were identified in the CCLE cohort.

Data S9. LEPs were identified in tumor cells preserved in our laboratory.

Data S10. LEPs were identified in the SYSUCC cohort.

Data S11. The positive rate and median expression of LEPs were identified in the SYSUCC cohort.

Data S12. The sequences of Primers, shRNAs, and siRNAs.
