# Supplementary material for: The cryptic lncRNA-encoded microprotein TPM3P9 drives oncogenic RNA splicing and tumorigenesis
Source: Signal Transduct Target Ther. 2025 Jan 27;10:43. doi: 10.1038/s41392-025-02128-8 (PMC11770092; doi:10.1038/s41392-025-02128-8)

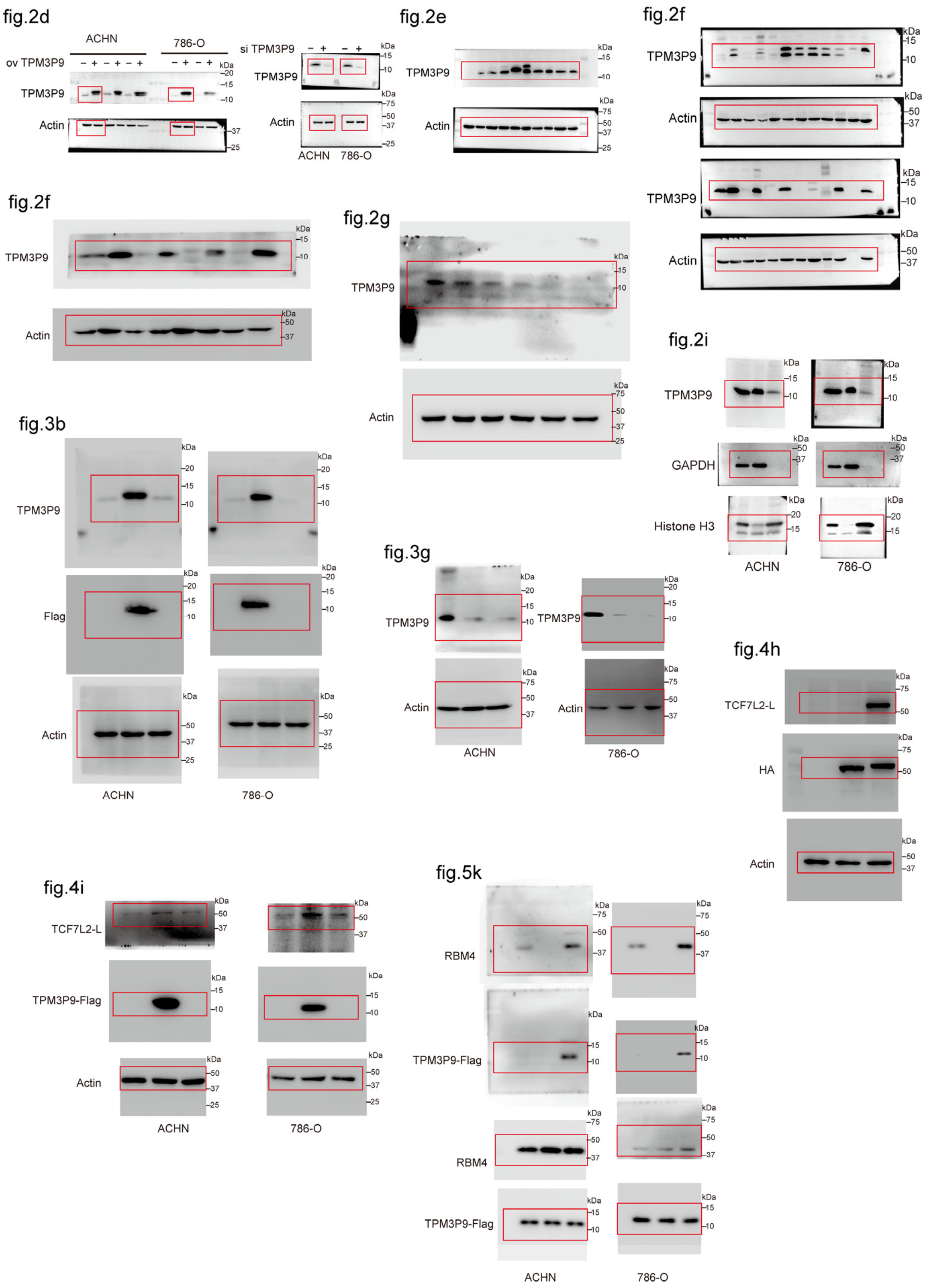
fig.2d
ACHN
786-O
ov TPM3P9
si TPM3P9
TPM3P9
Actin
ACHN 786-O
fig.2e
TPM3P9
Actin
fig.2f
TPM3P9
Actin
TPM3P9
Actin
fig.2f
TPM3P9
Actin
fig.2g
TPM3P9
Actin
fig.2i
TPM3P9
GAPDH
Histone H3
ACHN
786-O
fig.3b
TPM3P9
Flag
Actin
ACHN
786-O
fig.3g
TPM3P9
TPM3P9
Actin
Actin
ACHN
786-O
fig.4h
TCF7L2-L
HA
Actin
fig.4i
TCF7L2-L
TPM3P9-Flag
Actin
ACHN
786-O
fig.5k
RBM4
TPM3P9-Flag
RBM4
TPM3P9-Flag
ACHN
786-O

fig.5l

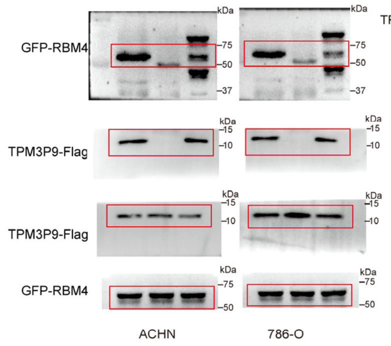

fig.5m

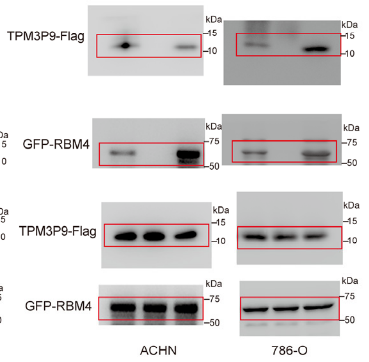

fig.6b

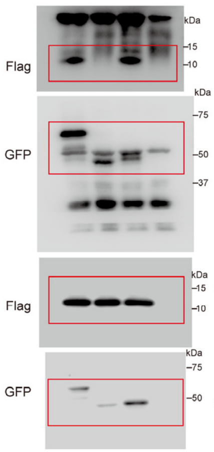

fig.6c

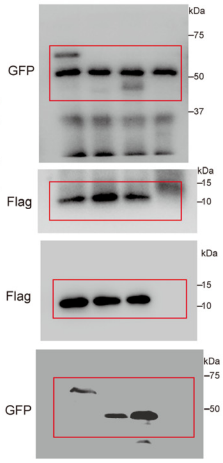

fig.6e

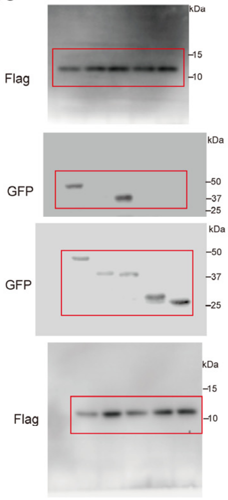

fig.6f

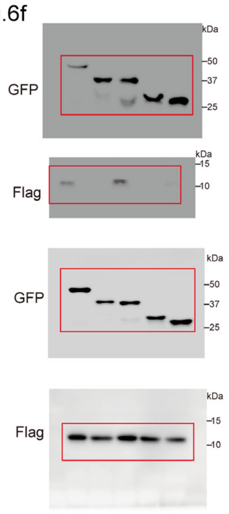

fig.7a

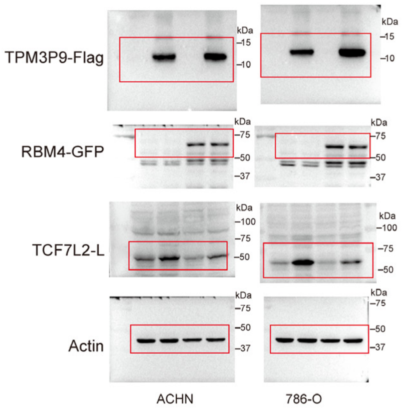

fig.7e

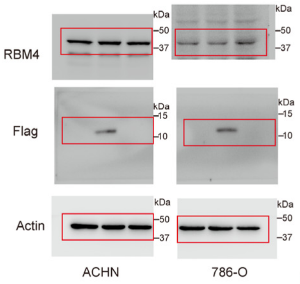

fig.7l

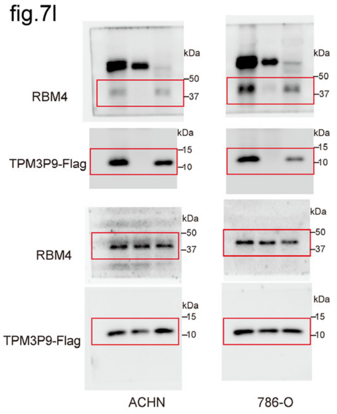

fig.7m

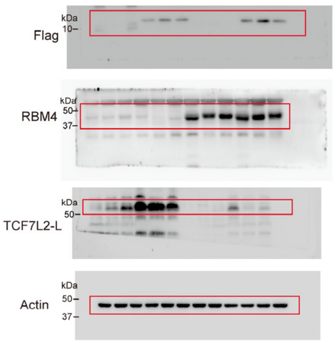

fig.8f

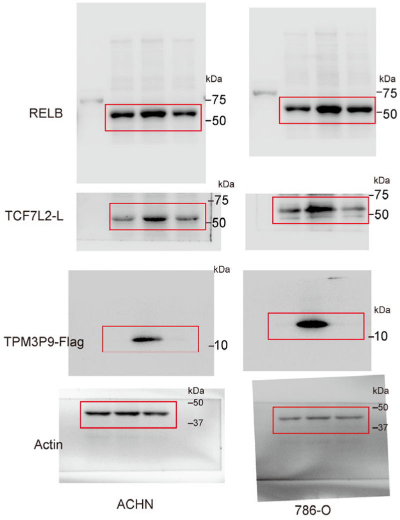

fig.8g

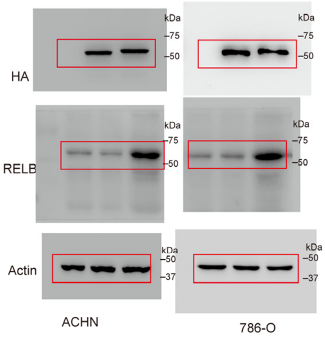

fig.8k

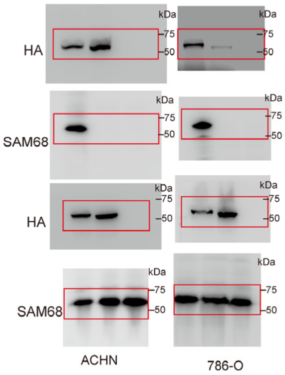

fig.8l

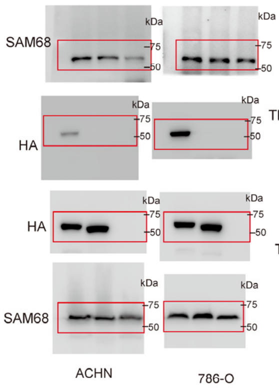

fig.8m

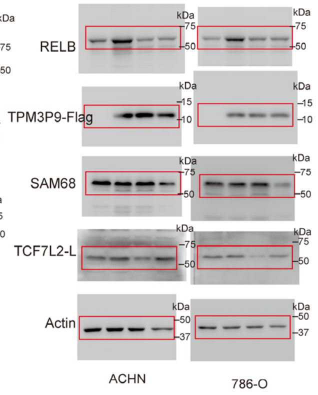

fig.S9b

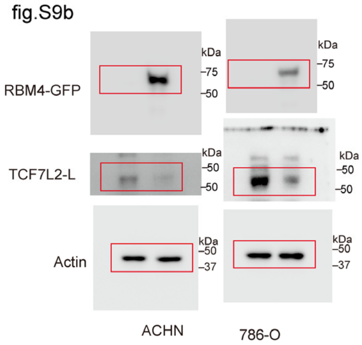

fig.S10a

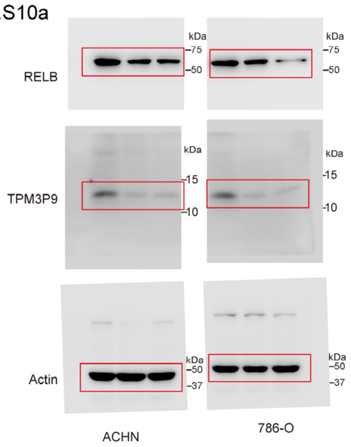

Supplement: Supplementary file 2 — western blot raw data [file 41392_2025_2128_MOESM2_ESM.pdf]
